# A phenome-wide association study (PheWAS) in the Population Architecture using Genomics and Epidemiology (PAGE) study reveals potential pleiotropy in African Americans

**DOI:** 10.1371/journal.pone.0226771

**Published:** 2019-12-31

**Authors:** Sarah A. Pendergrass, Steven Buyske, Janina M. Jeff, Alex Frase, Scott Dudek, Yuki Bradford, Jose-Luis Ambite, Christy L. Avery, Petra Buzkova, Ewa Deelman, Megan D. Fesinmeyer, Christopher Haiman, Gerardo Heiss, Lucia A. Hindorff, Chun-Nan Hsu, Rebecca D. Jackson, Yi Lin, Loic Le Marchand, Tara C. Matise, Kristine R. Monroe, Larry Moreland, Kari E. North, Sungshim L. Park, Alex Reiner, Robert Wallace, Lynne R. Wilkens, Charles Kooperberg, Marylyn D. Ritchie, Dana C. Crawford

**Affiliations:** 1 Genentech, Inc., South San Francisco, California, United States of America; 2 Department of Statistics, Rutgers University, Piscataway, New Jersey, United States of America; 3 Department of Genetics, Rutgers University, Piscataway, New Jersey, United States of America; 4 Illumina, Inc., San Diego, California, United States of America; 5 Department of Genetics, Institute for Biomedical Informatics, Perelman School of Medicine, University of Pennsylvania, Philadelphia, Pennsylvania, United States of America; 6 Information Sciences Institute; University of Southern California, Marina del Rey, California, United States of America; 7 Department of Epidemiology, University of North Carolina, Chapel Hill, North Carolina, United States of America; 8 Department of Biostatistics, University of Washington, Seattle, Washington, United States of America; 9 Amgen, Thousand Oaks, California, United States of America; 10 Department of Preventive Medicine, Keck School of Medicine, University of Southern California/Norris Comprehensive Cancer Center, Los Angeles, California, United States of America; 11 Carolina Center for Genome Sciences, University of North Carolina, Chapel Hill, North Carolina, United States of America; 12 National Human Genome Research Institute, National Institutes of Health, Bethesda, Maryland, United States of America; 13 Center for Research in Biological Systems, Department of Neurosciences, University of California, San Diego, La Jolla, California, United States of America; 14 The Ohio State University, Columbus, Ohio, United States of America; 15 Division of Public Health Sciences, Fred Hutchinson Cancer Research Center, Seattle, Washington, United States of America; 16 Epidemiology Program, University of Hawaii Cancer Center, Honolulu, Hawaii, United States of America; 17 University of Pittsburgh, Pittsburgh, Pennsylvania, United States of America; 18 Department of Epidemiology, University of Washington, Seattle, Washington, United States of America; 19 Departments of Epidemiology and Internal Medicine, University of Iowa, Iowa City, Iowa, United States of America; 20 Cleveland Institute for Computational Biology, Cleveland, Ohio, United States of America; 21 Departments of Population and Quantitative Health Sciences and Genetics and Genome Sciences, Case Western Reserve University, Cleveland, Ohio, United States of America; The University of North Carolina at Chapel Hill, UNITED STATES

## Abstract

We performed a hypothesis-generating phenome-wide association study (PheWAS) to identify and characterize cross-phenotype associations, where one SNP is associated with two or more phenotypes, between thousands of genetic variants assayed on the Metabochip and hundreds of phenotypes in 5,897 African Americans as part of the Population Architecture using Genomics and Epidemiology (PAGE) I study. The PAGE I study was a National Human Genome Research Institute-funded collaboration of four study sites accessing diverse epidemiologic studies genotyped on the Metabochip, a custom genotyping chip that has dense coverage of regions in the genome previously associated with cardio-metabolic traits and outcomes in mostly European-descent populations. Here we focus on identifying novel phenome-genome relationships, where SNPs are associated with more than one phenotype. To do this, we performed a PheWAS, testing each SNP on the Metabochip for an association with up to 273 phenotypes in the participating PAGE I study sites. We identified 133 putative pleiotropic variants, defined as SNPs associated at an empirically derived p-value threshold of p<0.01 in two or more PAGE study sites for two or more phenotype classes. We further annotated these PheWAS-identified variants using publicly available functional data and local genetic ancestry. Amongst our novel findings is *SPARC* rs4958487, associated with increased glucose levels and hypertension. *SPARC* has been implicated in the pathogenesis of diabetes and is also known to have a potential role in fibrosis, a common consequence of multiple conditions including hypertension. The *SPARC* example and others highlight the potential that PheWAS approaches have in improving our understanding of complex disease architecture by identifying novel relationships between genetic variants and an array of common human phenotypes.

## Introduction

Pleiotropy, however defined, has long been recognized as a feature of genomes with respect to their relationships to individual traits and outcomes that characterize phenomes [1–3]. Interest in human pleiotropy has spiked in the last decade owing to the availability of large genotype-phenotype datasets generated from genome-wide association studies (GWAS). The analysis and catalog collection of one phenotype versus many genotypes studies revealed that a sizable proportion of common genetic variants are associated with multiple related and independent phenotypes [4, 5]. These observations have led to the development of more systematic approaches to identify variant-level pleiotropy [6, 7], many of which have been applied to populations of mostly European-descent individuals ascertained in clinical settings (e.g., [8]).

Here, we describe a phenotype wide association study (PheWAS), a systematic approach to identify cross-phenotype associations, in the Population Architecture using Genomics and Epidemiology (PAGE) I study. The PAGE I study was established by the National Human Genome Research Institute (NHGRI) in 2008 with the intent to characterize GWAS-identified variants discovered in European populations using more diverse populations drawn from epidemiologic [9] and clinical [10] studies. The scope of the PAGE I study was subsequently expanded to include discovery and fine-mapping efforts using the Metabochip [11], a fixed-content array of ~200,000 variants designed to interrogate previously-identified GWAS variants as well as select genome regions related to cardio-metabolic traits for fine-mapping [12].

In this PheWAS, we investigated the associations between the 144,740 common genetic variants assayed on the Metabochip and 273 phenotypes collected in 5,897 African Americans participating in three epidemiologic PAGE I studies: the Atherosclerosis Risk in Communities (ARIC) [13]; Multiethnic Cohort (MEC) [14]; and the Women’s Health Initiative (WHI) [15]. We identified 133 potentially pleiotropic variants, defined as associated with two or more phenotype classes at p<0.01 in two or more PAGE I study sites. We functionally annotated PheWAS-identified variants and characterized the local genetic ancestry in this admixed population. From these data, we highlight variants likely to be pleiotropic and worthy of further statistical and functional studies. These data also underscore the necessity of diversity in study populations and study designs in PheWAS to ensure that all possible genotype-phenotype human relationships are considered.

## Results

**Fig 1:**
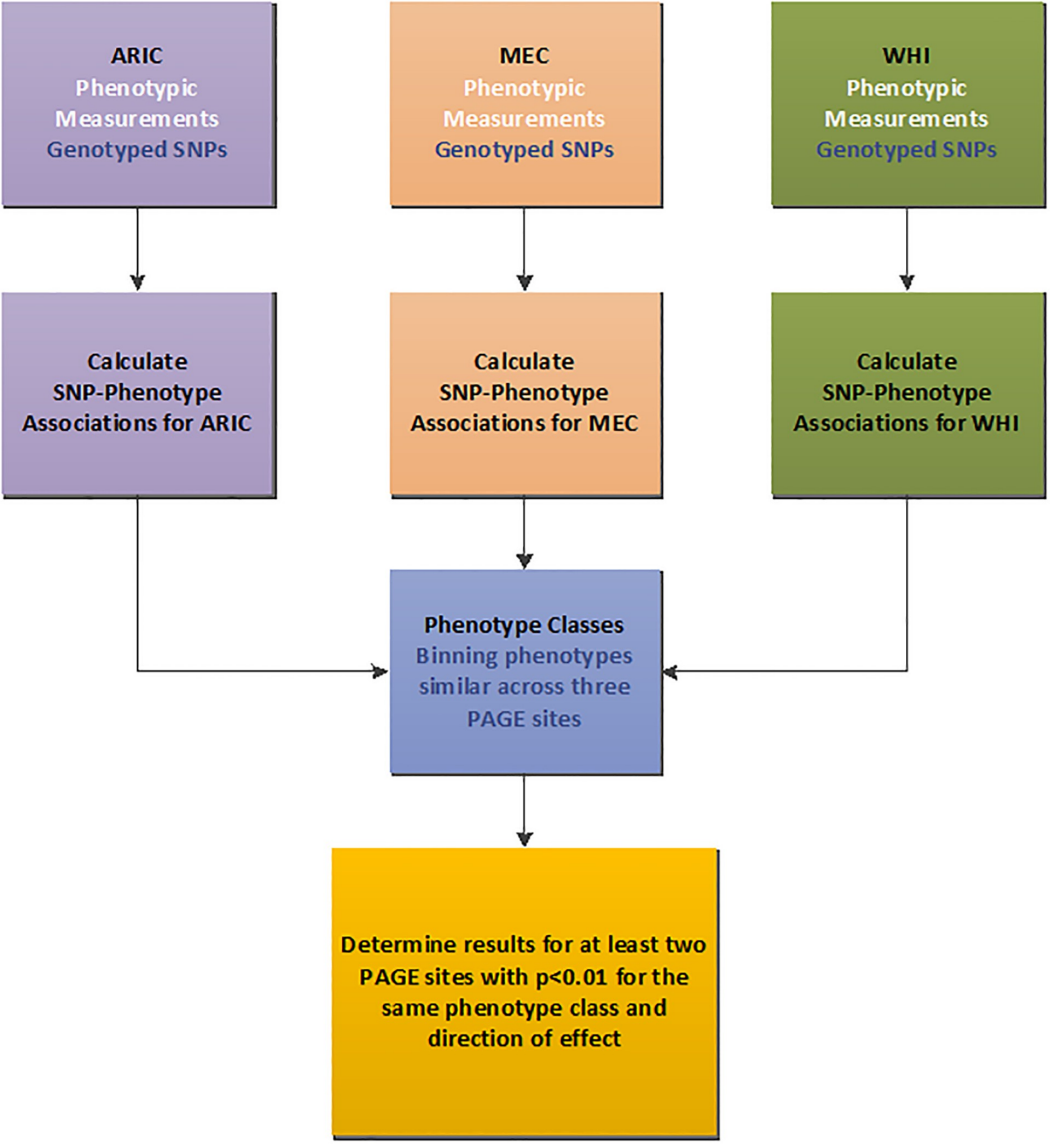
Overview of the Metabochip PheWAS study. Three different PAGE study sites contributed data to the project: Atherosclerosis Risk in Communities (ARIC); the Women’s Health Initiative (WHI), and the Multiethnic Cohort (MEC). Comprehensive tests of association between 144,740 Metabochip SNPs and 273 phenotypes were calculated for African American participants from each of the three PAGE study sites. Similar phenotypes that were collected across the studies were binned into “phenotype classes”. Our PheWAS-significant criteria required an association at p<0.01 in at least two PAGE study sites for the same phenotype class and direction of effect.

For this PheWAS (Fig 1), we comprehensively tested for associations between 114,740 SNPs assayed on the Metabochip with up to 273 phenotypes (S1 Table) available for 5,897 African American participants from three PAGE I studies: Atherosclerosis Risk in Communities (ARIC); Multiethnic Cohort (MEC); and the Women’s Health Initiative (WHI) (Table 1). Due to variations in the data collected across these epidemiologic studies, some phenotypes were available in more than one study, such as C-reactive protein (CRP) and low density lipoprotein cholesterol (LDL-C), while other phenotypes were only available within a single study, such as albumin level measurements. In **Methods** we describe further the studies included in this PheWAS, details of Metabochip genotyping and quality control, and the PheWAS approach including phenotype classification and filtering by statistical significance.

**Table 1:** Population Architecture using Genomics and Epidemiology (PAGE) I studies available for PheWAS and their characteristics. The Atherosclerosis Risk in Communities (ARIC), Multiethnic Cohort (MEC), and Women’s Health Initiative (WHI) from the PAGE I study contributed data towards this PheWAS. A full list of phenotypes used in this PheWAS is available in S1 Table. Some of the phenotypes were measured in more than one study, some phenotypes were related to phenotypes of another study, and some phenotypes were unique measurements for a single study. Not all phenotypic measurements were available for all participants within each study. Maximum sample size and minimum sample size are dependent both on which individuals were genotyped and which individuals also had a specific phenotype measured. See Materials and methods for more information.

Study	Age Range (in years)	Sex	Number of Phenotypes	Maximum Sample Size	Minimum Sample Size
ARIC	45–64	Males and Females	98	3,430	47
MEC	45–75	Males and Females	43	549	14
WHI	50–79	Females only	121	2,186	13

### Replication of previously described genotype-phenotype associations

**Fig 2:**
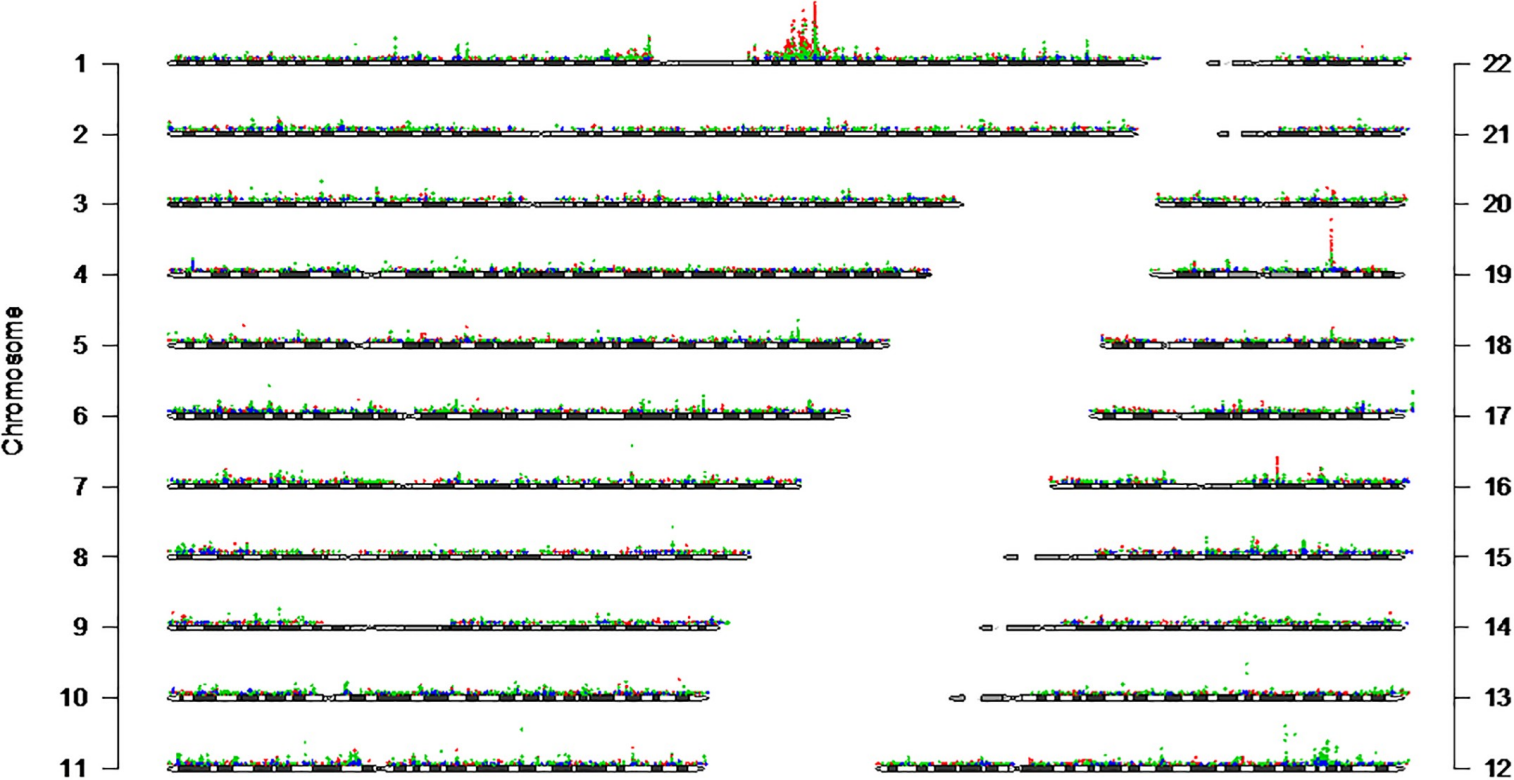
All genetic tests of association results, by PAGE study. Results from all tests of association for SNPs with a minor allele frequency >1% regardless of phenotype at p<5x10^-4^ were plotted across chromosomes 1–22. Each dot on the plot represents the −log10(p-value) for the test of the association, and each of the three PAGE studies is plotted with a different color, Multiethnic Cohort (MEC) in blue, Women’s Health Initiative (WHI) in green, and Atherosclerosis Risk in Communities (ARIC) in red. The most significant p-value plotted here is 8.01E-44 for *OLFML2B* rs6676438 and (natural log) white blood count in ARIC (see Table 2). The y-axis for each chromosome is the −log10(p-value), and the x-axis is chromosomal base pair location.

**Fig 3:**
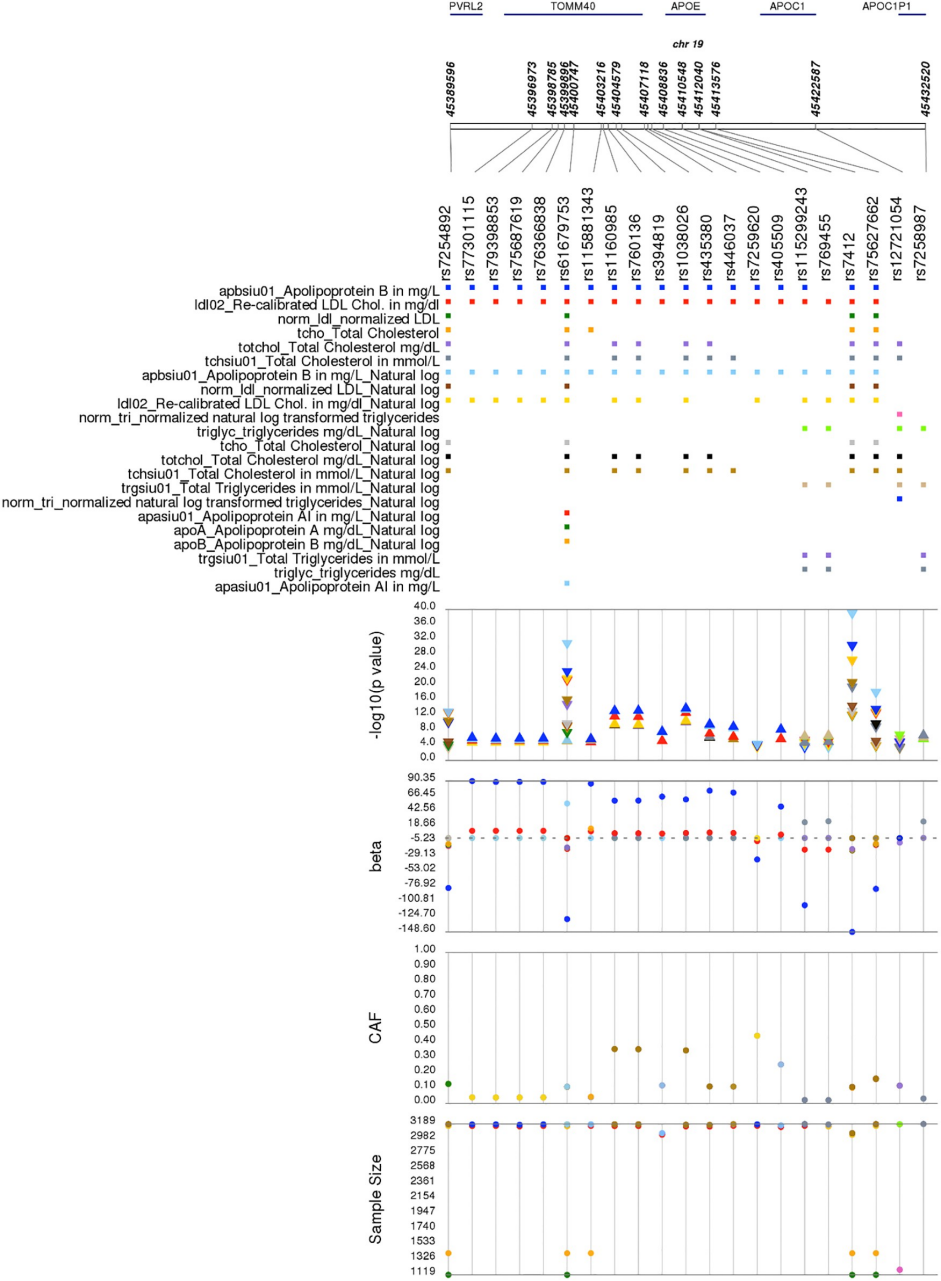
*APOE* rs7412 and nearby single nucleotide polymorphisms associated with lipid-related traits in a single Population Architecture using Genomics and Epidemiology (PAGE) study. Plotted are single SNP tests of association in the Atherosclerosis Risk in Communities (ARIC) for *APOE* rs7412 and nearby SNPs within 100kb of previously-reported genome-wide association study (GWAS) associations also associated here at p<1.0x10^-4^ with lipid-related traits. Data shown are sample size, coded allele frequency (CAF), genetic effect size (beta), -log10(p-value), and ARIC phenotypes on the y-axes. Each SNP is plotted on the x-axis at the top of the figure from 5´ to 3′, and genomic positions along chromosome 19 along with annotated genes are given above. Data are color-coded by phenotype and displayed as a square (for SNPs), a triangle (p-values), or closed circles (betas, CAFs, and sample size). Direction of the triangle represents the direction of the effect size.

We first performed comprehensive single SNP tests of associations for each PAGE I study across all SNPs with a minor allele frequency >1% on the Metabochip that passed quality control and all phenotypes available (Fig 2). Of note are the two association peaks on chromosomes 1 and 19. These peaks represent two previously known genotype-phenotype associations, and their identification here attests to the quality of this high-throughput PheWAS approach. The first association peak on chromosome 1 between *OLFML2B* rs6676438 and natural log-transformed white blood cell count (Table 2) recapitulates a known association in African Americans along this chromosomal region. *OLFML2B* rs6676438 is located on the short arm of chromosome 1 in a 90MB region known to be in linkage disequilibrium with the Duffy null allele (*DARC* rs2814778) and associated with hematological traits in African Americans [16]. The second most significant association peak on chromosome 19 (Fig 2) represents the known association between *APOE* rs7412 and natural log-transformed apolipoprotein B (Table 2) [17–19]. Apolipoprotein B is the primary apolipoprotein of LDL-C, a phenotype heavily scrutinized by candidate gene, GWAS, and sequencing studies. From these studies, *APOE* rs7412 is known to be associated with LDL-C in multiple populations [20–27] including European Americans [18, 19, 28–30] and African Americans [18, 19, 28, 30–32] as well as with related phenotypes such as response to statin therapy [33–37], small dense LDL-C [38], and lipid metabolism phenotypes for LDL-C and free cholesterol [39]. In the present PheWAS, *APOE* rs7412, along with nearby SNPs, were within 100kb of previously-reported GWAS associations and associated with the following lipid-related traits in a single PAGE I study (at p<1.0x10^-4^): total cholesterol, LDL-C, response to statin therapy, lipid metabolism phenotypes, and hypertriglyceridemia (Fig 3).

**Table 2:** Most significant and previously reported genotype-phenotype associations identified in the Population Architecture using Genomics and Epidemiology (PAGE) I study. Presented are the two most significant individual-level results for this PheWAS, along with other highly significant results for the same phenotype with a different transformation and/or study where the phenotype was available. Abbreviations: allele frequency (AF), standard error (SE).

SNP (Gene)	Phenotype	Phenotype Transformation	Study	P-value	Beta (SE)	Sample Size	Coded Allele	AF
rs6676438 (*OLFML2B*)	White blood count	Natural log	ARIC	8.01E-44	0.14 (0.01)	3,212	G	0.12
rs6676438 (*OLFML2B*)	White blood count	None	ARIC	4.17E-37	0.96 (0.08)	3,212	G	0.12
rs6676438 (*OLFML2B*)	White blood count	Natural log	WHI	8.99E-25	0.12 (0.01)	2,087	G	0.16
rs6676438 (*OLFML2B*)	White blood count	None	WHI	9.57E-06	3.38 (0.76)	2,087	G	0.16
rs7412 (*APOE*)	Apolipoprotein B (mg/L)	Natural log	ARIC	1.84E-40	-0.18 (0.01)	3,061	A	0.11
rs7412 (*APOE*)	Apolipoprotein B (mg/L)	None	ARIC	5.87E-32	-148.6 (12.49)	3,061	A	0.11

**Fig 4:**
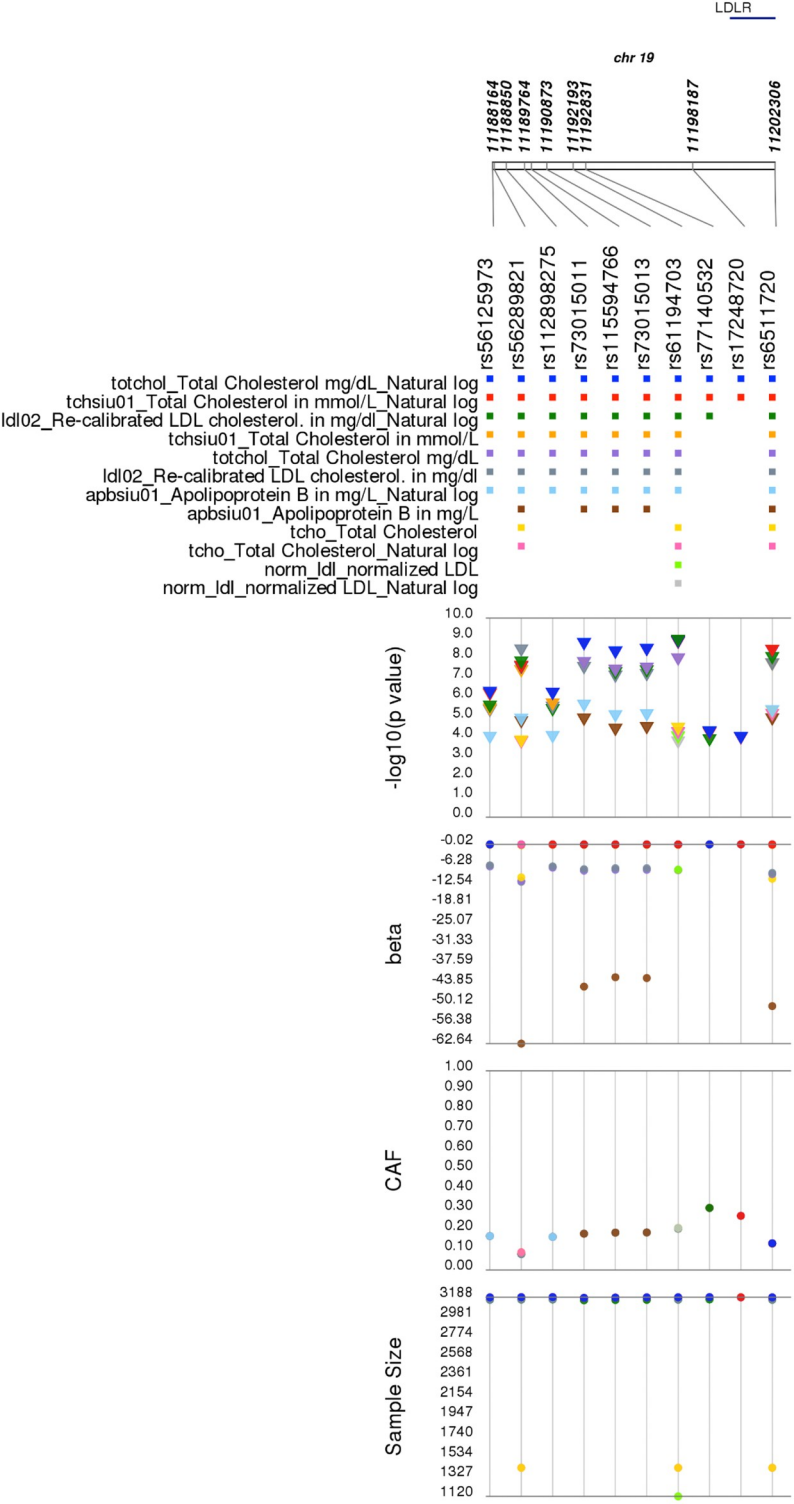
*LDLR* rs6511720 and nearby single nucleotide polymorphisms associated with lipid-related traits in a single Population Architecture using Genomics and Epidemiology (PAGE) study. Plotted are single SNP tests of association in the Atherosclerosis Risk in Communities (ARIC) for *LDLR* rs6511720 and nearby SNPs within 100kb of previously-reported genome-wide association study (GWAS) associations also associated here at p<1.0x10^-4^ with lipid-related traits. Data shown are sample size, coded allele frequency (CAF), genetic effect size (beta), -log10(p-value), and ARIC phenotypes on the y-axes. Each SNP is plotted on the x-axis at the top of the figure from 5´ to 3′, and genomic positions along chromosome 19 along with annotated genes are given above. Data are color-coded by phenotype and displayed as a square (for SNPs), a triangle (p-values), or closed circles (betas, CAFs, and sample size). Direction of the triangle represents the direction of the effect size.

**Fig 5:**
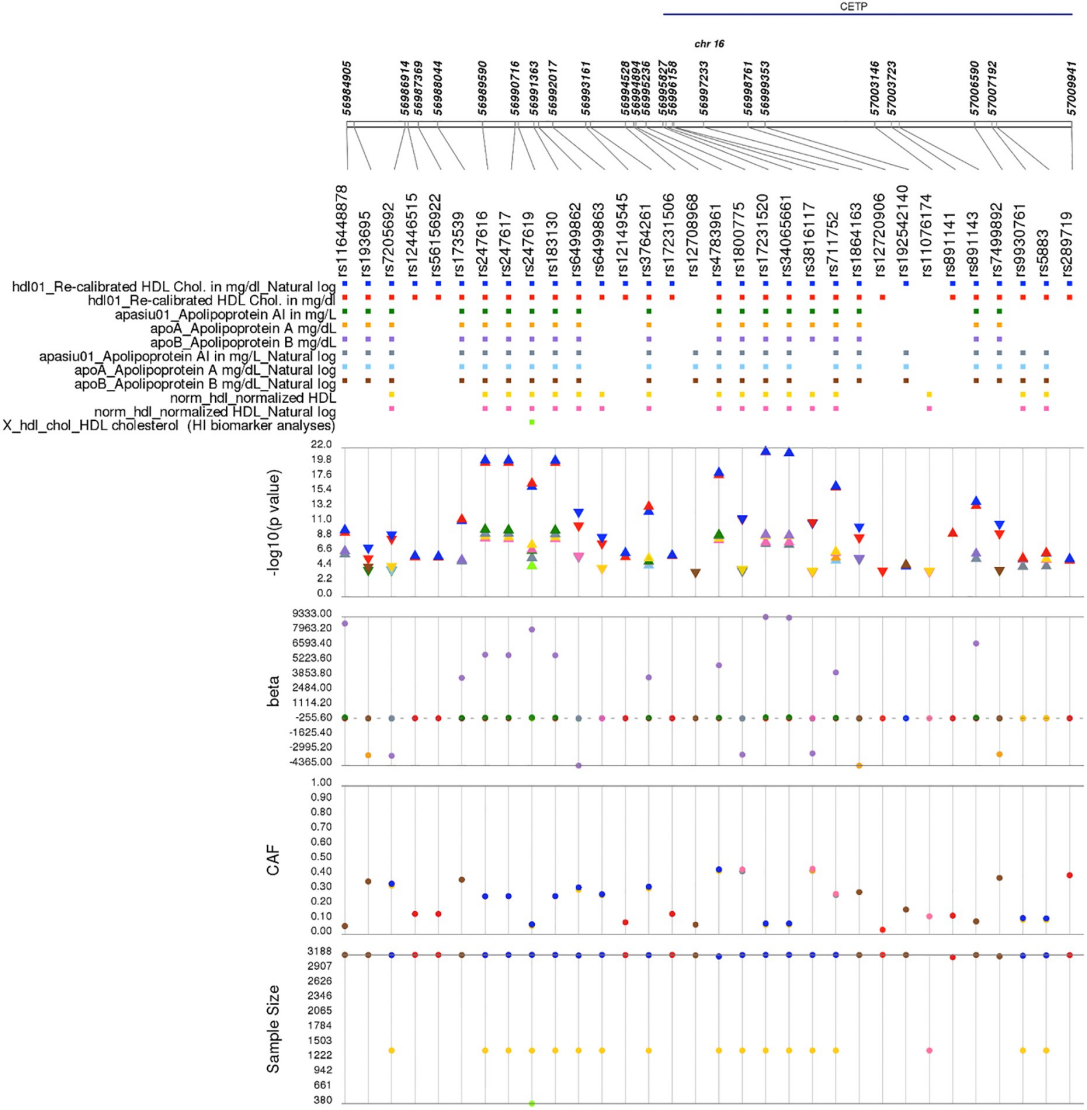
*CETP* rs3764261 and nearby single nucleotide polymorphisms associated with lipid-related traits in a single Population Architecture using Genomics and Epidemiology (PAGE) study. Plotted are single SNP tests of association in the Atherosclerosis Risk in Communities (ARIC) for *CETP* rs3764261 and nearby SNPs within 100kb of previously-reported genome-wide association study (GWAS) associations also associated here at p<1.0x10^-4^ with lipid-related traits. Data shown are sample size, coded allele frequency (CAF), genetic effect size (beta), -log10(p-value), and ARIC phenotypes on the y-axes. Each SNP is plotted on the x-axis at the top of the figure from 5´ to 3′, and genomic positions along chromosome 16 along with annotated genes are given above. Data are color-coded by phenotype and displayed as a square (for SNPs), a triangle (p-values), or closed circles (betas, CAFs, and sample size). Direction of the triangle represents the direction of the effect size.

In addition to the strongly associated chromosome 1 and 19 peaks, this PheWAS replicated other previously-reported GWAS findings. For example, *LDLR* rs6511720 was significantly associated with lipid measurements, including LDL-C (p = 1.13x10^-08^, beta(SE) = -9.0(1.60) in ARIC) (Fig 4), which has been reported in previous GWAS and genetic association studies in European Americans and African Americans [20, 40–43] where the A allele is associated with lower LDL-C levels. Likewise, the *CETP* rs3764261 was associated with HDL-C levels in African Americans (p = 1.13x10^-13^, beta(SE) = 3.48(0.47) in ARIC; Fig 5), as previously reported [20, 40, 43].

### Evidence of pleiotropy in African Americans

A total of 5,424 tests of association were significant at p<0.01 in two or more PAGE I studies and in the same direction for the same phenotype (S2 Table). To facilitate the identification of potential pleiotropy in African Americans, we grouped similar phenotypes measured in PAGE I studies into 30 phenotype classes regardless of genetic associations (Methods and Table 3). A PheWAS-identified variant then represented a variant associated with two or more phenotype classes meeting the significance threshold (Methods and S1 File). After phenotype class binning, we noted 133 SNPs associated with two or more distinct phenotype classes with the same direction of effect within a given phenotype class (S3 Table). As expected, the phenotype class combination ‘LDL-C/total cholesterol levels’ was associated with dozens (53) of the same SNPs. Also, 37 SNPs were associated with white blood count (WBC) coupled with other phenotype classes on chromosome 1, results likely driven by the Duffy polymorphism [16, 44].

**Table 3:** Phenotype classes represented in the Population Architecture using Genomics and Epidemiology (PAGE) I study PheWAS in African Americans. Phenotype-class binning first groups the phenotypes into categories within a PAGE study and then groups those categories across PAGE study sites. Below are the 30 phenotype classes that individually-labeled phenotypes across the studies were binned.

Activity Levels, Personal	Heart Arterial Surgery	Menarche
Alcohol Use	Heart Failure	Myocardial Infarction
Body Mass Index	Heart Rate	Platelet
Creatinine Levels	Height	Smoking
C-Reactive Protein Levels	Hematocrit	Stroke
Diabetes	Hemoglobin	Systolic Blood Pressure
Diastolic Blood Pressure	Hormone Use	Total Cholesterol
Fibrinogen	Hypertension	Triglycerides
Glucose Levels	Insulin	Weight
HDL-Cholesterol	LDL-Cholesterol	White Blood Count

**Fig 6:**
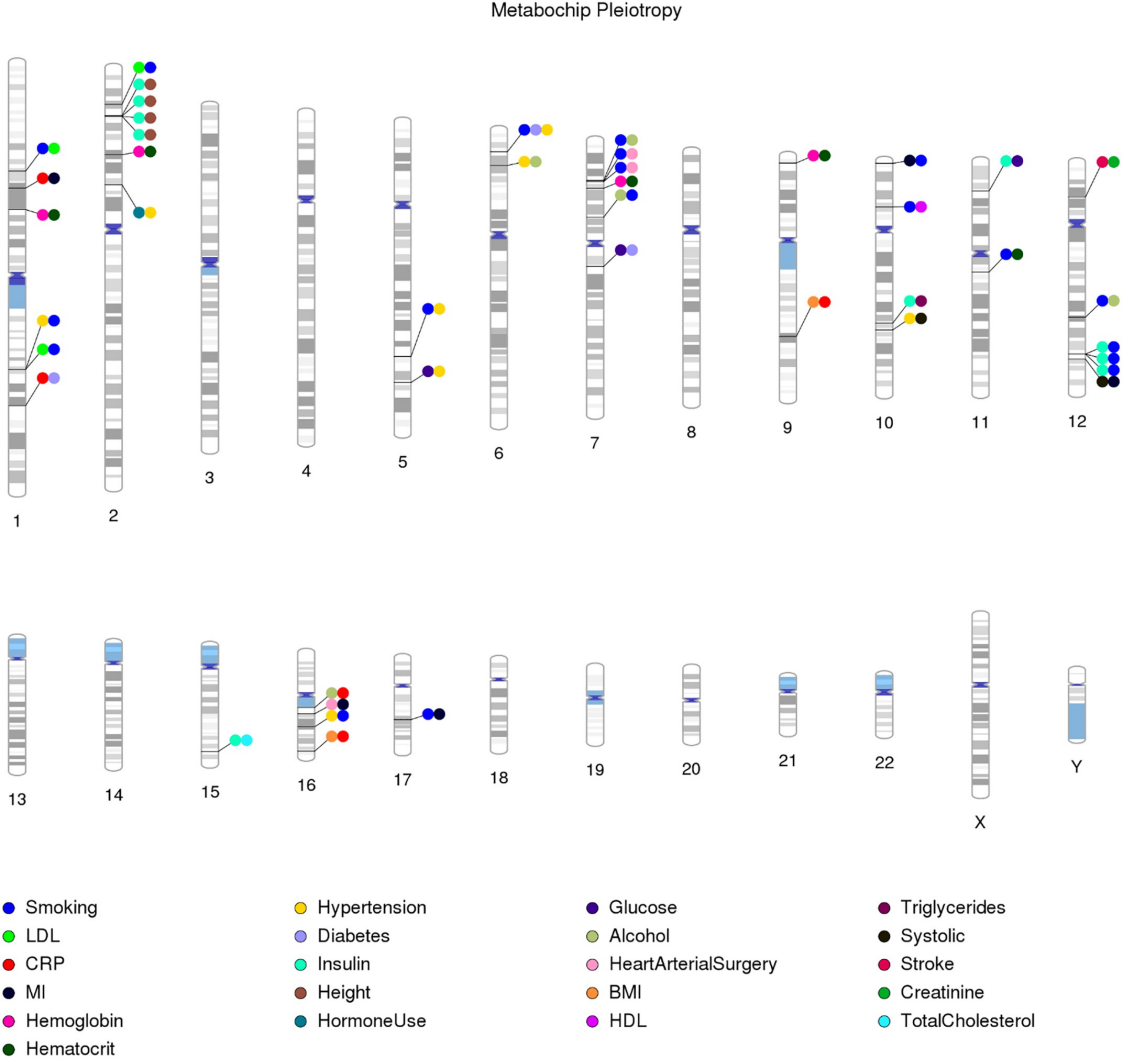
Phenotype classes associated with the same single nucleotide polymorphism in African Americans from the Population Architecture using Genomics and Epidemiology (PAGE) study. A plot of the 43 PheWAS results where two or more phenotype classes were significantly associated (p<0.01) with the same single nucleotide polymorphism (SNP) in participating PAGE study sites. This plot does not include concomitant lipid phenotype-class results (53 SNPs) or white blood cell related results for chromosome 1 (37 SNPs). Lines connect the SNP chromosomal location to circles, and the color of each circle corresponds to an associated phenotype class listed in the legend at the bottom.

The remaining 43 PheWAS-identified associations (Table 4; Fig 6) represent 38 independent associations at r^2^≥0.80 based on African population data from the 1000 Genomes Project [45]. Of these, seven (18.4%) PheWAS-identified variants were associated in the opposite direction between phenotype classes. Approximately half (20) of the phenotype-class combinations were associated with a single variant; the remainder were associated with more than one variant (Table 4). These multiple-associated phenotype classes were associated with two (insulin/height, body mass index/C-reactive protein, smoking/myocardial infarction, hypertension/smoking) and three (smoking/LDL-C, hemoglobin/hematocrit, smoking/alcohol consumption) variants each. One PheWAS-identified variant (rs9349379) was associated with three phenotype classes (smoking/diabetes/hypertension; Table 4 and Fig 6).

**Table 4:** Concomitant PheWAS results in African Americans from the Population Architecture using Genomics and Epidemiology (PAGE) study. Test of association results (p-values, betas, standard errors) are shown for variants associated at p-value <0.01 (empirically defined) with two or more phenotype classes. An asterisk in the direction of effect column indicates an opposite direction of effect for two of the phenotype classes listed. Genomic position given is based on hg38. Allele frequency is based on all African Americans across PAGE studies regardless of phenotype.

DE	rsID	Chromosome: position	Phenotype classes	PAGE Study	Phenotype	P-Value	Beta(se)	Sample size	CA	AF
	rs10889334	Chr1: 62491528	Smoking, LDL-C	WHI, WHI, WHI, WHI, WHI, ARIC, MEC, WHI	Years a regular smoker, Normalized LDL, Natural Log normalized LDL, Smoking—current (Y/N), Pack years of smoking, LN+1 RE-CALIBRATED LDL CHOL. in mg/dl, LN+1 SMKQUIT, Number of years since quit smoking (reported at baseline), LN+1 years a regular smoker	3.35e-3, 3.63e-3, 4.53e-3, 5.45e-3, 6.19e-3, 7.18e-3, 7.74e-3, 8.57e-3	-1.96e-1 (0.07), -4.73e0 (1.62), -3.71e-2 (0.01), -2.92e-1 (0.11), -1.20e0 (0.44), -2.37e-2 (8.82e-3), 0.10 (0.04), -6.52e-2 (0.02)	2029, 1120, 1120, 2186, 2017, 3162, 230, 2029	C	0.34
	rs61771778	Chr1: 72461172	CRP, MI	MEC, MEC, MEC, WHI, WHI, WHI	C-Reactive Protein, LN+1 C-Reactive Protein, Heart attack reported at baseline (Y/N), Age at MI, LN+1 CRP, LN+1 F2 age at MI	1.81e-4, 4.08e-4, 1.90e-3, 3.75e-3, 6.25e-3, 9.06e-3	2.13 (0.56), 0.42 (0.12), 1.23 (0.40), 2.37 (0.73), 0.26 (0.09), 0.56 (0.20)	380, 380, 455, 27, 1071, 27	G	0.043
	rs2994429	Chr1: rs2994429	Hemoglobin, Hematocrit	WHI, WHI, ARIC, WHI, WHI, ARIC	LN+1 Hemoglobin, Hemoglobin, LN+1 HEMATOCRIT, Hematocrit, LN+1 Hematocrit, LN+1 HEMOGLOBIN	5.83e-5, 1.09e-4, 2.17e-4, 8.82e-4, 9.40e-4, 2.76e-3	0.01 (2.54e-3), 0.14 (0.04), 9.29e-3 (2.51e-3), 0.36 (0.11), 8.65e-3 (2.61e-3), 7.43e-3 (2.48e-3)	2086, 2086, 3213, 2087, 2087, 3213	A	0.32
	rs10798572	Chr1: 177821975	Hypertension, Smoking	MEC, ARIC, WHI, ARIC	High blood pressure reported at baseline, CIGARETTE YEARS OF SMOKING, Smoking—current (Y/N), Blood Pressure Lowering Medications in the past 2 weeks—Took medication	1.62e-3, 6.90e-3, 8.74e-3, 9.27e-3	-4.71e-1 (0.15), -3.08e1 (11.41), -3.17e-1 (0.12), -1.51e-1 (0.06)	455, 3234, 2186, 3430	G	0.27
	rs943763	Chr1: 177867129	LDL-C, Smoking	WHI, WHI, WHI, MEC, MEC, ARIC, ARIC, ARIC	LN+1 normalized LDL, normalized LDL, Smoking—Former (Y/N), Pack-years of smoking (cigarettes) reported at baseline, LN+1 Pack-years of smoking (cigarettes) reported at baseline, Cigarette smoking status—Former smoker, LN+1 RE-CALIBRATED LDL CHOL. in mg/dl, HAVE YOU EVER SMOKED CIGARETTES? (Y/N)	6.59e-4, 9.64e-4, 2.12e-3, 3.46e-3, 5.13e-3, 6.29e-3, 7.99e-3, 8.13e-3	0.05 (0.01), 5.50 (1.66), 0.20 (0.06), 0.35 (0.12), 0.09 (0.03), -1.62e-1 (0.06), 0.02 (8.71e-3), -1.37e-1 (0.05)	1120, 1120, 2186, 435, 435, 3426, 3159, 3333	C	0.46
	rs1052238	Chr1: 198665496	CRP, Diabetes	WHI, ARIC, ARIC, ARIC, WHI	LN+1 CRP, C-reactive Protein, LN+1 C-reactive Protein, Diabetes—lower threshold 126 mg/dL—YES, Age first told had diabetes	3.25e-4, 2.63e-3, 7.85e-3, 8.70e-3, 9.70e-3	-0.13 (0.04), 3.12 (1.02), 0.27 (0.10), 0.17 (0.06), 0.25 (0.10)	1071, 150, 150, 3429, 207	G	0.45
	rs568938	Chr2: 21080744	LDL-C, Smoking	ARIC, ARIC, ARIC, MEC, ARIC, MEC. MEC	RE-CALIBRATED LDL CHOL. in mg/dl, LN+1 RE-CALIBRATED LDL CHOL. in mg/dl, LN+1 Smoking duration, Smoking duration (years) reported at baseline, Smoking duration, LDL cholesterol, LN+1 Smoking duration (years) reported at baseline	7.14e-7, 2.53e-6, 1.11e-3, 7.44e-3, 8.82e-3, 9.11e-3, 9.15e-3	5.44 (1.10), 0.04 (8.68e-3), 0.06 (0.02), 0.32 (0.12), 1.04 (0.40), 8.75 (3.34), 0.12 (0.05)	3162, 3162, 1751, 443, 1753, 380, 443	A	0.42
*	rs6722366	Chr2: 27809087	Insulin, Height	WHI, ARIC, ARIC, ARIC, ARIC, MEC, MEC	Insulin, LN+1 STANDING HEIGHT TO NEAREST CM, STANDING HEIGHT TO NEAREST CM, INSULIN (UU-ML), INSULIN in pmol/L, LN+1 Height (cm), Height (cm)	2.91e-7, 3.71e-4, 4.44e-4, 1.71e-3, 1.71e-3, 4.83e-3, 5.31e-3	23.15 (4.50), -1.29e-2 (3.62e-3), -2.16e0 (0.61), 10.25 (3.27), 73.53 (23.42), -3.13e-2 (0.01), -5.43e0 (1.94)	1718, 3332, 3332, 3243, 244, 451, 451	C	0.027
*	rs56197751	Chr2: 27835291	Insulin, Height	WHI, ARIC, ARIC, ARIC, ARIC, MEC, MEC	Insulin, LN+1 STANDING HEIGHT TO NEAREST CM, STANDING HEIGHT TO NEAREST CM, INSULIN (UU-ML), INSULIN in pmol/L, LN+1 Height (cm), Height (cm)	2.60e-7, 6.78e-4, 8.48e-4, 2.35e-3, 2.35e-3, 4.83e-3, 5.31e-3	23.72 (4.59), -1.24e-2 (3.65e-3), -2.07e0 (0.62), 9.92 (3.26), 71.15 (23.37). -3.13e-2 (0.01), -5.43e0 (1.94)	1718, 3266, 3266, 3179, 3179, 451, 451	A	0.028
*	rs114117339	Chr2: 27855059	Insulin, Height	WHI, ARIC, ARIC, ARIC, ARIC, MEC, MEC	Insulin, LN+1 STANDING HEIGHT TO NEAREST CM, STANDING HEIGHT TO NEAREST CM, INSULIN (UU-ML), INSULIN in pmol/L, LN+1 Height (cm), Height (cm)	2.60e-7, 7.45e-4, 9.16e-4, 1.28e-3, 1.28e-3, 4.83e-3, 5.31e-3	23.72 (4.59), -1.23e-2 (3.64e-3), -2.05e0 (0.62), 10.61 (3.29), 76.15 (23.63), -3.13e-2 (0.01), -5.43e0 (1.94)	1718, 3332, 3332, 3244, 3244, 451, 451	A	0.027
*	rs6760908	Chr2: 27855874	Insulin, Height	WHI, ARIC, ARIC, ARIC, ARIC, MEC, MEC	insulin, LN+1 STANDING HEIGHT TO NEAREST CM, INSULIN (UU-ML), INSULIN in pmol/L, STANDING HEIGHT TO NEAREST CM, LN+1 Height (cm), Height (cm)	2.60e-7, 7.53e-4, 8.32e-4, 8.32e-4, 9.27e-4, 4.83e-3, 5.31e-3	23.72 (4.59), -1.23e-2 (3.64e-3), 10.79 (3.23), 77.42 (23.14), -2.05e0 (0.62), -3.13e-2 (0.01), -5.43e0 (1.94)	1718, 3330, 3242, 3242, 3330, 451, 451	A	0.027
	rs12622858	Chr2: 50130369	Hemoglobin, Hematocrit	WHI, WHI, ARIC, WHI, ARIC, WHI, ARIC	Hemoglobin, LN+1 Hemoglobin, LN+1 HEMOGLOBIN, Hematocrit, HEMOGLOBIN, LN+1 Hematocrit, LN+1 HEMATOCRIT	3.45e-4, 2.13e-3, 2.63e-3, 4.98e-3, 6.00e-3, 7.24e-3, 7.77e-3	0.15 (0.04), 9.01e-3 (2.93e-3), 8.37e-3 (2.78e-3), 0.35 (0.13), 0.12 (0.05), 8.09e-3 (3.01e-3), 7.50e-3 (2.82e-3)	2086, 2086, 3206, 2087, 3206, 2087, 3206	A	0.21
	rs17033788	Chr2: 67566015	Hormone Use, Hypertension	ARIC, MEC, ARIC, WHI, WHI, ARIC, WHI	EVER TAKEN FEMALE HORMONES?, Age started estrogen: reported at baseline, Blood Pressure Lowering Medications in the past 2 weeks—Took medication, Hypertension ever (Y/N), Age told of hypertension, Never used hormones, LN+1 Age told of hypertension	2.60e-3, 3.52e-3, 5.60e-3, 6.36e-3, 6.66e-3, 6.84e-3, 7.03e-3	-5.16e-1 (0.17), 1.91 (0.60), -3.26e-1 (0.12), -3.51e-1 (0.13), -3.97e-1 (0.15), 0.44 (0.16), -1.43e-1 (0.05)	2064, 35, 3429, 2059, 2048, 3429, 2048	C	0.059
*	rs13186242	Chr5: 136857921	Smoking, Hypertension	ARIC, ARIC, ARIC, ARIC, ARIC, WHI, ARIC, WHI, WHI, WHI, WHI, WHI, WHI, ARIC, WHI, WHI	LN+1 CIGARETTE YEARS OF SMOKING, Blood Pressure Lowering Medications in the past 2 weeks—Took medication, Blood Pressure Lowering Medications in the past 2 weeks—Did not, Cigarette smoking status—Never smoker, HAVE YOU EVER SMOKED CIGARETTES? (Y/N), Age told of hypertension, CIGARETTE YEARS OF SMOKING, Never hypertensive (Y/N), LN+1 Age told of hypertension, Pack years of smoking, LN+1 Pack years of smoking, Smoking—Never (Y/N), Hypertension ever (Y/N), AGE 1ST REGULARLY SMOKED CIGARETS, Smoking—Former (Y/N), Treated hypertensive	3.94e-4, 5.74e-4, 1.19e-3, 1.33e-3, 1.61e-3, 3.49e-3, 4.18e-3, 4.30e-3, 4.85e-3, 4.96e-3, 5.92e-3, 7.42e-3, 7.85e-3, 8.30e-3, 9.77e-3, 9.94e-3	0.29 (0.08), -1.94e-1 (0.06), 0.18 (0.06), -1.84e-1 (0.06), 0.18 (0.06), -2.37e-1 (0.08), 31.35 (10.94), 0.20 (0.07), -8.32e-2 (0.03), 1.38 (0.49), 0.13 (0.05), -1.86e-1 (0.07), -1.88e-1 (0.07), -6.30e-1 (0.24), 0.18 (0.07), -1.82e-1 (0.07)	3231, 3427, 3427, 3427, 3334, 2047, 3231, 2185, 2047, 2016, 2016, 2185, 2058, 1763, 2185, 2185	A	0.28
	rs4958487	Chr5: 151684113	Glucose, Hypertension	ARIC, ARIC, ARIC, ARIC, WHI, WHI	LN+1 DERIVED GLUCOSE VALUE in mg/dl, HIGH BP EVER DIAGNOSED?, DERIVED GLUCOSE VALUE in mg/dl, HYPERTENSION,DEFINITION 5, Age told of hypertension, LN+1 glucose	1.29e-3, 6.65e-3, 6.65e-3, 6.71e-3, 7.21e-3, 8.38e-3	0.03 (8.14e-3), 0.14 (0.05), 3.92 (1.44), 0.14 (0.05), 0.20 (0.07), 0.02 (7.65e-3)	3245, 3301, 3245, 3325, 2048, 2000	A	0.40
*	rs9349379	Chr6: 12903725	Smoking, Diabetes, Hypertension	MEC, WHI, ARIC, WHI, WHI, WHI, MEC, ARIC, WHI	Ever smoke? (reported at baseline) (Y/N), LN+1 F33 Age started treatment for diabetes, HIGH BP EVER DIAGNOSED?, Age started treatment for diabetes, Smoking—Never (Y/N), Smoking—Former (Y/N), Diabetes reported at baseline (Y/N), Diabetes—lower threshold 126 mg/dL—YES, Treated hypertensive (Y/N)	1.69e-3, 2.35e-3, 2.50e-3, 2.62e-3, 2.68e-3, 5.61e-3, 5.73e-3, 6.94e-3, 9.01e-3	0.84 (0.27), 0.03 (0.01), -2.75e-1 (0.09), 2.13 (0.70), -3.09e-1 (0.10), 0.29 (0.10), -1.20e0 (0.43), -3.24e-1 (0.12), -2.73e-1 (0.10)	451, 668, 3301, 668, 2186, 2186, 455, 3430, 2186	G	0.099
	rs79239785	Chr6: 20602709	Alcohol, Hypertension	WHI, WHI, WHI, WHI, MEC, ARIC, ARIC	LN+1 Age told of hypertension, Hypertension ever (Y/N), Never hypertensive (Y/N), Age told of hypertension, Ethanol Drinks Per Day—<1, Drinker Status—Current drinker (Y/N), Blood Pressure Lowering Medications in the past 2 weeks—Took medication	3.84e-3, 4.55e-3, 5.22e-3, 6.26e-3, 7.26e-3, 9.28e-3, 9.72e-3	0.26 (0.09), 0.67 (0.24), -6.70e-1 (0.24), 0.69 (0.25), 1.39 (0.52), 0.47 (0.18), 0.44 (0.17)	2048, 2059, 2186, 2048, 549, 3430, 3430	A	0.020
	rs1728312	Chr7: 23231308	Smoking, Alcohol	WHI, WHI, WHI, MEC, ARIC, WHI	Age quit smoking, LN+1 Age quit smoking, LN+1 alcohol intake, Ethanol Drinks Per Day—<1, LN+1 AGE 1ST REGULARLY SMOKED CIGARETTES, alcohol intake	3.12e-3, 4.74e-3, 5.73e-3, 6.41e-3, 7.83e-3, 8.38e-3	0.86 (0.29), 0.11 (0.04), -7.38e-2 (0.03), 0.86 (0.31), -6.12e-2 (0.02), -2.70e-1 (0.10)	760, 760, 2060, 549, 1763, 2060	G	0.050
	rs2390859	Chr7: 23995928	Smoking, Heart arterial surgery	MEC, MEC, ARIC, MEC, MEC, ARIC, WHI, ARIC, MEC	Ever smoke? (reported at baseline) (Y/N), LN+1 Smoking duration (years) reported at baseline, AGE STOPPED SMOKING CIGARETTES, Smoking duration (years) reported at baseline, LN+1 Pack-years of smoking (cigarettes) reported at baseline, LN+1 AGE STOPPED SMOKING CIGARETTES, LN+1 Coronary bypass surgery ever (Y/N), Coronary Bypass surgery? Yes, Pack-years of smoking (cigarettes) reported at baseline	3.69e-3, 4.01e-3, 4.14e-3, 5.76e-3, 6.24e-3, 8.19e-3, 8.30e-3, 8.38e-3, 9.00e-3	0.66 (0.23), 0.18 (0.06), 2.09 (0.73), 0.44 (0.16), 0.12 (0.04), 0.05 (0.02), 0.96 (0.36), 0.87 (0.33), 0.42 (0.16)	451, 443, 771, 443, 435, 771, 2089, 3429, 435	A	0.17
	rs4722315	Chr7: 24006654	Smoking, Heart arterial surgery	MEC, MEC, MEC, MEC, WHI, ARIC, MEC, ARIC	LN+1 Smoking duration (years) reported at baseline, Smoking duration (years) reported at baseline, Ever smoke? (reported at baseline) (Y/N), LN+1 Pack-years of smoking (cigarettes) reported at baseline, Coronary bypass surgery ever (Y/N), Coronary Bypass surgery? Yes, Pack-years of smoking (cigarettes) reported at baseline, AGE STOPPED SMOKING CIGARETTES	4.84e-3, 5.61e-3, 6.82e-3, 7.05e-3, 7.54e-3, 7.71e-3, 8.77e-3, 8.95e-3	0.18 (0.06), 0.44 (0.16), 0.61 (0.23), 0.12 (0.04), 0.98 (0.37), 0.88 (0.33), 0.43 (0.16), 1.91 (0.73)	443, 443, 451, 435, 2089, 3429, 435, 771	G	0.17
	rs2106922	Chr7: 27969614	Hemoglobin, Hematocrit	WHI, WHI, WHI, ARIC, WHI, ARIC	LN+1 Hemoglobin, Hemoglobin, LN+1 Hematocrit, Hematocrit, Hematocrit, Hematocrit	4.70e-5, 2.84e-4, 1.55e-3, 2.08e-3, 3.74e-3, 8.17e-3	-0.012(2.84e-3), -0.15(0.04), -9.25e-3(2.92e-3), -0.14(0.05), -0.354(0.12), -0.36(0.13)	2086, 2086, 2087, 3212, 2087, 3212	A	0.22
	rs114374279	Chr7: 44532122	Alcohol, Smoking	ARIC, WHI, WHI, MEC, MEC	Drinker Status—Current drinker, LN+1 years a regular smoker, years a regular smoker, Ethanol Drinks Per Day—<2, LN+1 Number of years since quit smoking (reported at baseline)	1.38e-3, 5.02e-3, 5.54e-3, 9.28e-3, 9.67e-3	0.52 (0.16), -2.53e-1 (0.09), -6.77e-1 (0.24), 1.49 (0.57), -3.48e-1 (0.13)	3424, 2029, 2029, 549, 230	G	0.020
	rs11983880	Chr7: 73708374	Glucose, Diabetes	WHI, ARIC, WHI, WHI, ARIC, ARIC, WHI	LN+1 glucose, LN+1 DERIVED GLUCOSE VALUE in mg/dl, glucose, Diabetes ever, Diabetes—lower threshold 126 mg/dL—YES, DERIVED GLUCOSE VALUE in mg/dl, treated diabetes	1.05e-3, 1.47e-3, 1.48e-3, 3.02e-3, 3.07e-3, 4.39e-3, 7.35e-3	0.04 (0.01), 0.04 (0.01), 5.50 (1.73), 0.45 (0.15), 0.29 (0.10), 6.80 (2.39), 0.29 (0.11)	2000, 3186, 2000, 2086, 3370, 3186, 2089	A	0.10
	rs10974448	Chr9: 4308010	Hematocrit, Hemoglobin	WHI, ARIC, WHI, WHI, WHI, ARIC, ARIC	Hemoglobin, LN+1 HEMOGLOBIN, LN+1 Hemoglobin, LN+1 Hematocrit, Hematocrit, LN+1 HEMATOCRIT, HEMOGLOBIN	2.04e-3, 2.12e-3, 2.18e-3, 4.14e-3, 5.98e-3, 7.13e-3, 8.65e-3	0.14 (0.04), 8.95e-3 (2.91e-3), 9.20e-3 (3.00e-3), 8.83e-3 (3.08e-3), 0.35 (0.13), 7.93e-3 (2.95e-3), 0.12 (0.05)	2086, 3212, 2086, 2087, 2087, 3212, 3212	A	0.20
	rs2756916	Chr9: 101915669	BMI, CRP	ME, MEC, WHI, MEC, WHI, WHI, MEC	Body mass index (BMI) calculated from weight & height at baseline, LN+1 BMI Body mass index (BMI) calculated from weight & height at baseline, CRP, LN+1 C-Reactive Protein, LN+1 Body mass index (BMI) calculated from weight & height at baseline, Body mass index (BMI) calculated from weight & height at baseline, C-Reactive Protein	1.28e-3, 1.98e-3, 2.94e-3, 4.21e-3, 5.07e-3, 6.40e-3, 8.65e-3	3.28 (1.01), 0.11 (0.03), 5.20 (1.75), 0.49 (0.17), 0.05 (0.02), 1.61 (0.59), 2.17 (0.82)	445, 445, 107, 380, 2062, 2062, 380	C	0.026
	rs7923036	Chr10: 1433616,	MI, Smoking	ARIC, ARIC, WHI, MEC, MEC, ARIC	LN+1 age at first heart attack, Age at first heart attack, MI, LN+1 Number of years since quit smoking (reported at baseline), Number of years since quit smoking (reported at baseline), Number of years abstained from cigarettes	2.04e-4, 7.47e-4, 1.06e-3, 1.57e-3, 3.50e-3, 6.89e-3	-0.12 (0.03), -4.78 (1.38), 0.59 (0.18), 0.15 (0.05), 0.64 (0.22), 0.92 (0.34)	109, 109, 2089, 230, 230, 511	G	0.19
	rs787037	Chr10: 26504353	Smoking, HDL-C	WHI, WHI, ARIC, ARIC	Smoking—Never, normalized HDL, Smoking duration, RE-CALIBRATED HDL CHOL. in mg/dl	9.75e-4, 7.78e-3, 8.57e-3, 9.02e-3	-2.43e-1 (0.07), -3.06e-2 (0.01), 0.06 (0.02), -2.44e-2 (9.35e-3)	2186, 1383, 1745, 3181	G	0.21
	rs17875327	Chr10: 92515052	Insulin, Triglycerides	WHI, WHI, WHI, ARIC, ARIC, ARIC, ARIC, ARIC	insulin, normalized natural log transformed triglycerides, LN+1 normalized natural log transformed triglycerides, LN+1 INSULIN (UU-ML), TOTAL TRIGLYCERIDES in mmol/L, triglycerides mg/dL, LN+1 INSULIN in pmol/L, LN+1 TOTAL TRIGLYCERIDES in mmol/L	3.63e-5, 1.25e-3, 1.72e-3, 5.62e-3, 5.68e-3, 5.68e-3, 7.05e-3, 7.39e-3	21.55 (5.20), 0.17 (0.05), 0.03 (9.35e-3), 0.21 (0.07), 0.24 (0.09), 20.86 (7.54), 0.21 (0.08), 0.07 (0.03)	1718, 1190, 1190, 3244, 3189, 3189, 3244, 3189	G	0.018
	rs942008	Chr10: 96397655	Hypertension, SBP	ARIC, WHI, WHI, WHI, ARIC, WHI, ARIC, WHI, WHI, ARIC, ARIC, WHI	HIGH BP EVER DIAGNOSED?, Never hypertensive (Y/N), Treated hypertensive (Y/N), Hypertension ever (Y/N), Blood Pressure Lowering Medications in the past 2 weeks—Took medication, Age told of hypertension, Blood Pressure Lowering Medications in the past 2 weeks—Did not take, LN+1 systolic blood pressure, systolic blood pressure, 2ND AND 3RD SYSTOLIC BP AVERAGE, LN+1 2ND AND 3RD SYSTOLIC BP AVERAGE, Age told of hypertension	2.39e-4, 4.58e-4, 8.00e-4, 1.13e-3, 1.66e-3, 2.61e-3, 3.06e-3, 3.25e-3, 4.01e-3, 4.40e-3, 5.55e-3, 6.22e-3	0.24 (0.07), -2.90e-1 (0.08), 0.27 (0.08), 0.27 (0.08), 0.20 (0.06), 0.10 (0.03), -1.89e-1 (0.06), 0.02 (5.33e-3), 2.04 (0.71), 1.89 (0.66), 0.01 (4.85e-3), 0.26 (0.09)	3301, 2186, 2186, 2059, 3430, 2048, 3430, 2089, 2089, 3337, 3337, 2048	G	0.18
	rs76394293	Chr11: 17372388	Insulin, Glucose	WHI, WHI, ARIC, ARIC, ARIC, WHI	insulin, glucose, INSULIN (UU-ML), DERIVED GLUCOSE VALUE in mg/dl, INSULIN in pmol/L, glucose	1.07e-3, 3.63e-3, 5.05e-3, 5.48e-3, 5.63e-3, 8.39e-3	13.74 (4.19), 8.78 (3.01), 0.15 (0.05), 10.75 (3.87), 0.15 (0.06), 0.06 (0.02)	1718, 2000, 3244, 3245, 3244, 2000	A	0.032
	rs1939120	Chr11:64537243	Smoking, Hematocrit	WHI, ARIC, WHI, MEC, WHI	Smoking—Former, LN+1 HEMATOCRIT, Hematocrit, Currently smoke? (reported at baseline) (Y/N), LN+1 Hematocrit	2.72e-3, 4.96e-3, 8.14e-3, 8.85e-3, 9.24e-3	0.20 (0.07), 6.70e-3 (2.38e-3), 0.28 (0.11), 0.48 (0.18), 6.57e-3 (2.52e-3)	2186, 3210, 2087, 450, 2087	A	0.37
	rs17376366	Chr12: 20339790	Stroke, Creatinine	WHI, WHI, MEC, ARIC, ARIC	Age stroke, LN+1 Age stroke, Urinary Creatinine, New Maternal History of Stroke (Y/N), CREATININE (MG-DL)	2.38e-3, 3.10e-3, 7.60e-3, 9.20e-3, 9.31e-3	6.94 (2.20), 0.10 (0.03), 24.66 (9.17), 0.33 (0.13), 0.10 (0.04)	76, 76, 274, 3042, 3244	G	0.076
	rs115487129	Chr12: 89395852	Smoking, Alcohol	WHI, MEC, ARIC, WHI, ARIC	Smoking—current (Y/N), Ethanol Drinks Per Day—2+ drinks, Smoking duration, alcohol intake, LN+1 Smoking duration	1.44e-3, 3.60e-3, 4.97e-3, 8.34e-3, 9.14e-3	0.61 (0.19), 1.15 (0.39), -2.44e0 (0.87), 0.27 (0.10), -1.12e-1 (0.04)	2186, 549, 1753, 2061, 1751	C	0.052
*	rs7139221	Chr12: 110853890	Insulin, Smoking	WHI, ARIC, ARIC, ARIC, WHI	insulin, LN+1 AGE STOPPED SMOKING CIGARETTES, AGE STOPPED SMOKING CIGARETTES, LN+1 INSULIN (UU-ML), Pack years of smoking	1.47e-3, 3.12e-3, 4.14e-3, 6.04e-3, 9.28e-3	6.97 (2.19), -6.23e-2 (0.02), -2.37e0 (0.82), 0.07 (0.03), -1.70e0 (0.65)	1718, 771, 771, 3243, 2017	A	0.14
*	rs61944267	Chr12: 110856526	Insulin, Smoking	WHI, ARIC, ARIC, ARIC, WHI, ARIC	insulin, LN+1 AGE STOPPED SMOKING CIGARETTES, AGE STOPPED SMOKING CIGARETTES, LN+1 INSULIN (UU-ML), Pack years of smoking, LN+1 INSULIN in pmol/L	1.66e-3, 3.70e-3, 4.94e-3, 5.67e-3, 6.78e-3, 9.24e-3	6.82 (2.16), -6.13e-2 (0.02), -2.33e0 (0.83), 0.07 (0.03), -1.75e0 (0.65), 0.07 (0.03)	1718, 770, 770, 3242, 2017, 3242	G	0.14
*	rs113945414	Chr12: 110859018	Insulin, Smoking	WHI, ARIC, ARIC, ARIC, WHI, ARIC	Insulin, LN+1 AGE STOPPED SMOKING CIGARETTES, LN+1 INSULIN (UU-ML), AGE STOPPED SMOKING CIGARETTES, pack years of smoking, LN+1 INSULIN in pmol/L	1.45e-3, 3.46e-3, 4.42e-3, 4.54e-3, 6.80e-3, 7.31e-3	6.90 (2.16), -6.16e-2 (0.02), 0.08 (0.03), -2.34e0 (0.82), -1.75e0 (0.65), 0.08 (0.03)	1718, 771, 3243, 771, 2017, 3243	A	0.15
	rs10774711	Chr:113697994	SBP, MI	ARIC, WHI, WHI, ARIC, WHI, ARIC, WHI	2ND AND 3RD SYSTOLIC BP AVERAGE, LN+1 Age at MI, Age at MI, HEART ATTACK EVER DIAGNOSED?, LN+1 systolic blood pressure, LN+1 2ND AND 3RD SYSTOLIC BP AVERAGE, systolic blood pressure	3.37e-3, 3.61e-3, 4.10e-3, 4.25e-3, 5.86e-3, 6.78e-3, 7.41e-3	-1.53e0 (0.52), -4.92e-2 (0.02), -3.40e0 (1.15), -3.47e-1 (0.12), -1.11e-2 (4.03e-3), -1.04e-2 (3.83e-3), -1.44e0 (0.54)	3334, 77, 77, 3305, 2089, 3334, 2089	A	0.49
	rs60136502	Chr15: 90965307	Insulin, Total Cholesterol	WHI, MEC, ARIC, ARIC, ARIC, ARIC, WHI	insulin, LN+1 Insulin, TOTAL CHOLESTEROL in mmol/L, total cholesterol mg/dL, LN+1 TOTAL CHOLESTEROL in mmol/L, LN+1 total cholesterol mg/dL, total cholesterol	1.32e-3, 1.52e-3, 5.85e-3, 5.85e-3, 7.26e-3, 7.38e-3, 8.18e-3	7.38 (2.29), 0.22 (0.07), 0.13 (0.05), 4.99 (1.81), 0.02 (7.11e-3), 0.02 (8.44e-3), 6.48 (2.45)	1718, 442, 3182, 3182, 3182, 3182, 1417	C	0.11
	rs11865790	Chr16: 47399623	Alcohol, CRP	ARIC, WHI, ARIC, MEC, WHI	Drinker Status—Current drinker, LN+1 alcohol intake, LN+1 C-reactive Protein, LN+1 C-Reactive Protein, alcohol intake	2.65e-4, 5.57e-3, 7.59e-3, 7.78e-3, 9.31e-3	0.21 (0.06), -3.45e-2 (0.01), 0.31 (0.12), 0.15 (0.06), -1.24e-1 (0.05)	3428, 2061, 151, 380, 2061	G	0.35
	rs8058543	Chr16: 53096347	Heart Arterial Surgery, MI	WHI, ARIC, ARIC, ARIC, ARIC, ARIC, WHI	Percutaneous transluminal coronary angioplasty (Y/N), Heart or arterial surgery? No, MI by 2007 (Y/N), Heart or arterial surgery? Yes, Coronary Bypass surgery? No, LEFT HEART OR ART SURG—left leg, MI ever (Y/N)	3.00e-4, 5.32e-4, 9.00e-4, 1.21e-3, 3.90e-3, 5.43e-3, 6.32e-3	1.76 (0.49), -1.02e0 (0.30), 0.60 (0.18), 1.05 (0.32), 1.11 (0.38), 2.47 (0.89), 1.12 (0.41)	2044, 3429, 3339, 3429, 3429, 3429, 2088	A	0.044
*	rs2926143	Chr16: 64224173	Hypertension, Smoking	MEC, ARIC, WHI, WHI, ARIC, WHI, WHI	High blood pressure reported at baseline, HYPERTENSION DEFINITION 5, Never hypertensive (Y/N), Hypertension ever (Y/N), CIGARETTE YEARS OF SMOKING, LN+1 Age told of hypertension, Pack years of smoking	1.03e-3, 1.24e-3, 4.41e-3, 4.58e-3, 7.11e-3, 7.65e-3, 9.58e-3	-4.47e-1 (0.14), -1.59e-1 (0.05), -1.78e-1 (0.06), 0.18 (0.06), 26.25 (9.75), 0.07 (0.03), 1.13 (0.44)	455, 3325, 2186, 2059, 3234, 2048, 2017	G	0.49
	rs4260044	Chr16: 85238638	BMI, CRP	ARIC, ARIC, MEC, MEC, ARIC, MEC, MEC	BODY MASS INDEX IN KG/(M*M), LN+1 BODY MASS INDEX IN KG/(M*M), Body mass index (BMI) calculated from weight & height at baseline, LN+1 BMI Body mass index (BMI) calculated from weight & height at baseline, Log (CRP), LN+1 C-Reactive Protein, C-Reactive Protein	4.39e-4, 8.07e-4, 1.10e-3, 1.82e-3, 2.72e-3, 5.26e-3, 7.52e-3	0.54 (0.15), 0.02 (4.80e-3), 1.01 (0.31), 0.03 (0.01), 0.24 (0.08), 0.14 (0.05), 0.65 (0.24)	3327, 3327, 445, 445, 378, 380, 380	A	0.48
*	rs4334353	Chr17: 55374378	Smoking, MI	ARIC, ARIC, ARIC, MEC, ARIC, WHI, MEC	AGE 1ST REGULARLY SMOKED CIGARETS Q29, AGE 1ST REGULARLY SMOKED CIGARETS Q29, AGE AT FIRST HEART ATTACK, Number of years since quit smoking (reported at baseline), AGE AT FIRST HEART ATTACK, age at MI, Number of years since quit smoking (reported at baseline)	1.02e-4, 1.30e-3, 2.62e-3, 6.51e-3, 6.83e-3, 8.33e-3, 9.88e-3	0.04 (0.01), 0.77 (0.24), -9.08e-2 (0.03), 0.50 (0.18), -3.42e0 (1.24), -2.39e-1 (0.08), 0.10 (0.04)	1723, 1723, 109, 230, 109, 27, 230	A	0.32

Abbreviations: allele frequency (AF), C-reactive protein (CRP), coded allele (CA), direction of effect (DE), high density lipoprotein cholesterol (HDL-C), low density lipoprotein cholesterol (LDL-C), systolic blood pressure (SBP), myocardial infarction (MI), standard error (SE)

Apart from the expected pleiotropic associations represented by the LDL-total cholesterol and white blood cell phenotype classes, this PheWAS in African Americans from PAGE revealed potentially novel pleiotropic relationships, notably with phenotype classes that represent common exposure, lifestyle, or environmental variables. For example, rs568938 was associated with both LDL-C and smoking phenotype classes (Table 4 and Fig 6). The LDL-C/rs568938 association has been previously described in diverse populations [20, 46]; however, the association with the smoking phenotype class is novel regardless of population. The direction of effect for these associations suggests that the coded allele of rs568938 is associated with both increasing LDL-C and duration of smoking reported (Table 4), results that are consistent with epidemiological studies that describe a relationship between smoking and increased LDL-C [47]. Likewise, *DOCK7* rs10889334, previously associated with total cholesterol [48] and cardiovascular disease [49] via linkage disequilibrium, was also associated with LDL-C and smoking phenotype classes in the same direction (Table 4). Among the non-LDL-C associations, PheWAS-identified *PHACTR1* rs9349379 was associated with the three phenotype classes of smoking, diabetes, and hypertension in opposing directions. The *PHACTR1* association with hypertension in this PheWAS is supported by the recent GWAS literature for blood pressure [49–51]. In contrast, the opposite-direction-of-effect association observed for smoking and diabetes is not yet supported by genetic data but instead supported by some of the epidemiologic literature where those who report current smoking have lower blood pressure and less hypertension compared with non-smokers (e.g., [52]). Other exposure, lifestyle, and environmental phenotype classes implicated in this PheWAS include alcohol consumption and hormone use (Table 4).

### Functional and ancestral annotation of potentially pleiotropic SNPs

To better understand the functional impact of the 38 potentially pleiotropic SNPs (Table 4), we implemented several *in silico* annotation approaches for these as well as proxy SNPs (in linkage disequilibrium at r^2^≥0.8) using various public resources, including HaploReg v4.1 [53], RegulomeDB v1.1 [54], and the SNP and CNV Annotation Database (SCAN) [55]. Almost all (94.7%) of these 38 PheWAS-identified variants are intronic (20) or intergenic (16), with the remaining two classified as synonymous (rs114374279) and missense (rs76394293; Table 5). We note that most (24) PheWAS-identified variants were annotated as associated with gene expression or as expression quantitative trait loci (eQTL) in at least one resource used here (Table 5).

**Table 5:** Functional and ancestral annotation of potentially pleiotropic single nucleotide polymorphisms in the Population Architecture using Genomics and Epidemiology (PAGE) study. All PheWAS-identified variants and variants in linkage disequilibrium with the index variant (at r^2^ ≥ 0.80 based AFR 1000 Genomes Project data) were functionally and ancestrally annotated using HaploReg v4.1, RegulomeDB v1.1, the SNP and CNV Annotation Database (SCAN), and local genetic ancestry. HaploReg v4.1 NHGRI-EBI GWAS Catalog data were augmented using NHGRI-EBI GWAS Catalog data from 2019-06-20, and associations listed are at p<5.0x10^-8^ unless otherwise noted. Local genetic ancestry, shown as proportion of African-derived alleles, was estimated using LAMP-LD. An asterisk in the direction of effect column indicates an opposite direction of effect for two of the phenotype classes listed. Genomic position given is based on hg38.

DE	rsID (Chromsome:Position)	Phenotype classes	CA	Reason on Metabochip	Variant classification (Gene or nearest gene(s))	Variants in LD	HaploReg/RegulomeDB/SCAN	Ancestry		
								0	1	2
	rs10889334 (chr1:62491528)	Smoking, LDL-C	C	Fine-mapping Lipids / Triglycerides	Intronic (*DOCK7*)	rs10493322 rs10158897 rs3913007 rs4350231 rs12037659 rs11356503 rs1979722 rs10889333 rs10789112 rs10889335	HaploReg: located within enhancer and promoter regions in multiple cell types; index variant significant eQTL in GTEx transformed fibroblast cells, skin (sun exposed lower leg), tibial artery, and aorta artery tissues; *USP1* rs10158897 associated with LDL-C in Chinese population at p = 9.0x10^-6^ (PMID:24386095); *DOCK7* rs4350231 is associated with cardiovascular disease in European populations (PMID:30595370); *DOCK7* rs10889333 is associated with total cholesterol in European populations (PMID:28270201) RegulomeDB: rs4350231 likely to affect TF binding and linked to expression of a gene target (*DOCK7*); rs10493322, rs10158897, rs3913007, rs11356503, rs10889334 minimal TF binding evidence SCAN: Index variant an eQTL in multiple genes in CEU. Associated SNPs rs10493322, rs10158897, rs3913007, rs4350231, rs1979722, rs10889333, rs10889335 also eQTLs in multiple genes, mostly CEU data. Associated rs10889335, is a nonsynonymous enhancer	7.57	43.53	48.90
	rs61771778 chr1:72461172)	CRP, MI	G	Fine-mapping BMI	Intronic (*LOC105378797*)	rs4649955	HaploReg: Index and associated variants significant eQTLs in GTEx from tibial nerve tissue	42.34	52.41	5.24
	rs2994429 (chr1:84709901)	Hemoglobin, Hematocrit	A	Replication Fasting Glucose	Intergenic (*SSX2IP/ LPAR3*)	rs2911571 rs2911578 rs2994449 rs1340527 rs1340528	HaploReg: Index variant is an eQTL in whole blood; associated rs2911571 binds OCT2 and POU2F2 and has promoter and enhancer histone marks in several cell lines RegulomeDB: Associated variant rs2911571 likely to affect TF binding; index variant less likely to affect TF binding; other associated variants have minimal TF binding evidence SCAN: Index and associated variants have eQTL evidence in YRI cell line	100.00	0.00	0.00
	rs10798572 (chr1:177821975)	Hypertension, Smoking	G	Fine-mapping BMI	Intergenic (*LOC100506128/ SEC16B*)		HaploReg: associated with *HMGN1*, *PTGES2*, and *SNX29* gene expression in peripheral blood monocytes SCAN: eQTL evidence across multiple genes in YRI cell line	0.00	1.06	98.94
	rs943763 (chr1:177867129)	LDL-C, Smoking	C	Fine-mapping BMI	Intergenic (*LOC100506128/ SEC16B*)	rs3131318	RegulomeDB: Index variant has minimal TF binding evidence SCAN: Index variant is eQTL with multiple genes in YRI cell line	50.75	35.36	13.89
	rs1052238 (chr1:198665496)	CRP, Diabetes	G	Replication BMI	Intronic (*PTPRC*)	rs1926230 rs12401369 rs6686725 rs1326272 rs3820484 rs16843591 rs201584212 rs57114949 rs59147127 rs3767735 rs56272733 rs6428473 rs2182418 rs2148314 rs6428474 rs3754096 rs1326274 rs1326275 rs1326276 rs1052240	Haploreg: Index variant is eQTL for *PTPRC* in whole blood; associated rs2148314 is strong and weak enhancer in multiple cell lines. RegulomeDB: Index variant and associated variants rs6686725, rs6428474, rs1326272, rs56272733, rs3754096, rs1052240 have minimal TF binding evidence; associated variants rs1926230, rs2182418, rs2148314 less likely to affect TF binding	59.00	40.92	0.08
	rs568938 (chr2:21080744)	LDL-C, Smoking	A	Fine-mapping Lipids/LDL	Intergenic (*APOB/ LOC100129278*)	rs563290 rs668948 rs201027918 rs541041 rs478588 rs577584 rs614303 rs503105	HaploReg: Index and associated variants rs478588 *APOB* eQTL in QTEx heart-left ventricle tissue; associated variants are weak to strong enhancers in cell lines; multiple proteins bound to associated rs201027918 and rs503105; index and associated variants rs563290, rs668948, rs541041 associated with LDL-C, triglycerides, and total cholesterol in multiple populations (PMID:29507422); associated rs577584 associated with LDL-C in multiple populations (PMID:30275531); associated rs541041 associated with response to statin therapy in European-descent populations at p = 8x10^-6^ (PMID:20339536) RegulomeDB: Associated rs201027918 likely to affect TF binding; minimal TF binding evidence for associated rs503105, rs563290, rs577584 SCAN: Index variant and associated rs563290, rs668948, rs201027918, rs541041, rs478588, rs577584, rs614303, rs503105 eQTLs for several genes in CEU cell line	22.79	64.53	12.68
*	rs56197751 (chr2:27835291)	Insulin, Height	A	Fine-mapping Lipids/Triglycerides	Intronic (*RBKS*)		HaploReg: Strong and weak enhancer across multiple cell lines RegulomeDB: minimal TF binding evidence			
*	rs6760908 (chr2:27855874)	Insulin, Height	A	Fine-mapping Lipids/Triglycerides	Intronic (*RBKS*)	rs6722366 rs140932929 rs116259477 rs138952225 rs147803188 rs114117339 rs142916112 rs116767574 rs6752310 rs111456535 rs144150514 rs141914200 rs145123975	HaploReg: Index and associated variants strong and weak enhancers across multiple cell lines; associated rs138952225 is a missense variant RegulomeDB: Associated rs142916112 likely to affect TF binding; index variant and associated variants rs140932929, rs138952225, rs114117339, rs116767574, rs111456535, rs141914200, rs116259477, rs147803188 have minimal TF binding evidence	0.00	8.73	91.27
	rs12622858 (chr2:50130369)	Hemoglobin, Hematocrit	A	Replication Triglycerides	Intronic (*NRXN1*)	rs1452778 rs4971554 rs1452781 rs1452783 rs12477934 rs1961358 rs2011317 rs12474335 rs1377233 rs10490241 rs921573 rs1452768 rs930752 rs72878120 rs72878122 rs72878124 rs4971648 rs12469244 rs17039848 rs4971556 rs57366806 rs9917277 rs17039863 rs6744441 rs896683 rs896684 rs1880075 rs896685 rs1840074 rs11898302 rs11887484 rs12479057 rs1037428 rs72878159 rs12151722 rs12151727 rs11125288 rs11883844 rs12622858 rs12465888 rs12478732 rs17039935	HaploReg: Index variant *NRXN1* eQTL for GTEx testis tissue; associated variants strong enhancers across multiple cell lines RegulomeDB: Minimal TF binding evidence for associated rs17039863, rs1452778, rs72878124, rs4971648, rs896683, rs11898302, rs1452783, rs12477934, rs1377233, rs72878120, rs72878122, rs17039848, rs57366806, rs1880075, rs1037428, rs11125288, rs12478732 SCAN: Index and associated variants rs1452778, rs1452783, rs1961358, rs1377233, rs10490241, rs921573, rs930752, rs4971648, rs896683, rs896684, rs12478732 eQTLs for multiple genes in YRI and CEU cell lines	37.62	62.38	0.00
	rs17033788 (chr2:67566015)	Hormone Use, Hypertension	C	Replication Diastolic Blood Pressure	Intronic (*LOC102724373/LOC105374786*)	rs11686555 rs17033787 rs17033788 rs4671187 rs4671188 rs60735969 rs2270342 rs2270344 rs2270346 rs5831889 rs12624204 rs12616261 rs1107595 rs2270348 rs12619942 rs10490721 rs72621572 rs4671189 rs4671808 rs723712 rs4444509 rs11679082 rs150584652 rs2861650 rs2861651 rs149039938 rs148027161 rs143593647 rs2902020 rs11680926	HaploReg: Index and associated variants rs11686555, rs17033787, rs17033788, rs4671187, rs4671188, rs60735969, rs12619942, rs72621572, rs4671189, rs4671808, rs723712, rs11679082, rs143593647, rs2902020, rs11680926 eQTLs for GTEx testis tissue; index variant strong enhancer across multiple cell lines. RegulomeDB: Index variant and associated rs4671187, rs4671188, rs2270344, rs2270342, rs17033787 have minimal binding TF evidence; associated rs60735969 likely to affect TF binding	95.74	4.26	0.00
*	rs13186242 (chr5:136857921)	Smoking, Hypertension	A	Replication Fasting Glucose	Intergenic (*LOC391834*)			53.59	46.41	0.00
	rs4958487 (chr5:151684113)	Glucose, Hypertension	A	Replication Mean Platelet Volume	Intronic (*SPARC*)		HaploReg: Strong promoter and enhancer in several cell lines; eQTL for GTEx tibial artery and skeletal muscle tissues RegulomeDB: minimal TF binding evidence. SCAN: eQTL for several genes in CEU and YRI cell lines	62.82	37.12	0.06
*	rs9349379 (chr6:12903725)	Smoking, Diabetes, Hypertension	G	Fine-mapping Myocardial Infarction, Replication Diastolic Blood Pressure, Replication Systolic Blood Pressure	Intronic (*PHACTR1*)		HaploReg: Strong enhancer in several cell lines; eQTL for GTEx aorta artery, coronary artery, and tibial artery tissues; associated with coronary heart disease in European and South Asian populations (PMID:21378988; PMID:21846871), in a Lebanese population (PMID:22745674), and in Han Chinese (PMID:22751097); coronary artery calcification in European populations (PMID:22144573); cervical artery dissection in European populations (PMID:25420145); migraine (PMID:23793025) (PMID:22683712), migraine without aura (PMIS:2379025), and clinic-based migraine (PMID:23793025) in European populations; headache in British population (PMID:29397368); alcohol consumption in European-descent populations (PMID:30643258); systolic and diastolic blood pressure in multiple populations (PMID:27618447; PMID:30595370; PMID:30578418); pulse pressure in multiple populations (PMID:27618447; PMID:30578418; PMID:27841878) RegulomeDB: minimal TF binding evidence	13.30	29.44	57.26
	rs79239785 (chr6:20602709)	Alcohol, Hypertension	A	Fine-mapping Type 2 Diabetes	Intronic (*CDKAL1*)	rs116186936 rs182046163	HaploReg: Associated rs116186936 active promoter across multiple cell lines RegulomeDB: Index and associated variants have minimal TF binding evidence	100.00	0.00	0.00
	rs1728312 (chr7:23231308)	Smoking, Alcohol	G	Replication HDL	Intronic (*LOC101927890*)	rs75143479 rs199354	HaploReg: Index variant strong enhancer and promoter in multiple cell lines; Index and associated variants are eQTLs for GTEx whole blood; index variant and associated rs75143479 are eQTLs for GTEx subcutaneous adipose tissues; associated 199354 is a synonymous variant RegulomeDB: Associated rs199354 is less likely to affect TF binding	36.41	62.61	0.98
	rs2390859 (chr7:23995928)	Smoking, Heart arterial surgery	A		Intergenic (*STK31/ NPY*)	rs9655243 rs9655244 rs10447618 rs2390859 rs7783960 rs7784349 rs67876243 rs873186 rs1072098 rs10243572 rs10257228 rs70939867 rs6943764 rs6947634 rs7801000 rs2390860 rs7802938 rs202074308 rs7807099 rs112444386 rs4719751 rs4722315 rs6461766 rs6461767 rs4722316 rs4719752 rs4719753 rs6977179 rs12700507 rs7810381 rs7791318 rs7791476 rs199821255 rs11417764 rs12670613 rs12670671 rs7801241 rs7784401 rs7801557 rs6461771 rs6461772 rs7788937 rs7788943 rs6461773 rs6461774 rs6974395 rs6974276 rs6956710 rs6978673 rs6978761	HaploReg: Index variant promoter in one cell line; associated variants weak and strong enhancers and promoters in multiple cell lines RegulomeDB: Index and associated variants rs10243572, rs10447618, rs199821255, rs200555303, rs202074308, rs4719752, rs4722316, rs6461767, rs6461771, rs6461772, rs6461773, rs67876243, rs6943764, rs6956710, rs6974276, rs6974395, rs6977179, rs6978673, rs6978761, rs7801557, rs7802938, rs7807099, rs9655243, rs9655244 have minimal evidence for TF binding SCAN: Associated variants rs9655243, rs10447618, rs6943764, rs7802938, rs4719752, rs12700507, rs7791476, rs7801241, rs7801557, rs7788937, rs6461774, rs6974395 eQTLs for several genes in YRI cell line	36.22	62.66	1.12
	rs2106922 (chr7:27969614)	Hemoglobin, Hematocrit	A	Fine-mapping Type 2 Diabetes	Intronic (*JAZF1*)	rs17695685 rs34389716	HaploReg: Index and associated variants enhancers in several cell lines and eQTL in lymphoblastoid EUR cell lines RegulomeDB: Minimal TF binding evidence for associated rs17695685 and rs34389716 SCAN: Index and associated variant rs17695685 eQTLs for several genes in CEU and YRI cell line	100.00	0.00	0.00
	rs114374279 (chr7:44532122)	Alcohol, Smoking	G	Fine-mapping Lipids, LDL	Synonymous (*NPC1L1*)		HaploReg: Enhancer for several cell lines RegulomeDB: minimal TF binding evidence	0.00	4.35	95.65
	rs11983880 (chr7:73708374)	Glucose, Diabetes	A	Fine mapping Lipids/Triglycerides	Intronic (*STX1A*)	rs11973069	HaploReg: Index and associated rs11973069 enhancers for several cell lines RegulomeDB: Index and associated rs11973069 have minimal TF binding evidence	80.68	19.30	0.02
	rs10974448 (chr9:4308010)	Hematocrit, Hemoglobin	A	Fine Mapping Fasting Glucose	Intergenic (*GLIS3*)		HaploReg: Enhancer for several cell lines RegulomeDB: minimal TF binding evidence SCAN: eQTL for several genes in YRI cell line	5.18	66.94	27.89
	rs2756916 (chr9:101915669)	BMI, CRP	C		Intergenic (*GRIN3A/ ARL2BPP7*)	rs10448294 rs143133298 rs143437487 rs201788915 rs200346607 rs150008750 rs823919 rs823915 rs146165843 rs2756916 rs2795380 rs823911 rs10739817 rs2995211 rs3012590 rs2995212 rs2995215 rs78207849 rs147999115 rs149109326 rs139794562 rs149531772 rs143226478 rs181646361 rs144087235 rs140683088	HaploReg: Index variant associated with exon level expression of GRIN3A RegulomeDB: Index and associated variants rs146165843, rs2995212, rs78207849, rs143226478, rs10448294, rs2795380, rs823911, rs10739817, rs147999115, rs149109326, rs139794562, rs140683088 have minimal TF binding evidence SCAN: Associated variants rs823911, rs2995211, rs2995212 eQTLs for several genes in CEU cell line	99.52	0.48	0.00
	rs7923036 (chr10:1433616)	MI, Smoking	G	Replication BMI	Intronic (*ADARB2*)		HaploReg: Promoter and enhancer across multiple cell lines RegulomeDB: Minimal TF binding evidence	29.50	70.50	0.00
	rs787037 (chr10:26504353)	Smoking, HDL-C	G		Intronic (*APBB1IP*)		HaploReg: Promoter and enhancer across multiple cell lines RegulomeDB: Minimal TF binding evidence SCAN: eQTL for multiple genes in YRI cell line	17.56	81.95	0.49
	rs17875327 (chr10:92515052)	Insulin, Triglycerides	G	Fine-mapping Type 2 Diabetes	Intronic (*IDE*)	rs201503938	HaploReg: Index and associated variant *MARK2P9* eQTL in GTEx aorta artery, cerebellum brain, transformed fibroblast cells, muscularis esophagus, lung, skeletal muscle, pancreas, suprapubic skin not sun exposed, lower leg skin sun exposed, thyroid tissues RegulomeDB: Index and associated variant have minimal TF binding evidence	100.00	0.00	0.00
	rs942008 (chr10:96397655)	Hypertension, SBP	G	Replication Fasting Glucose	Intronic (TLL2)		HaploReg: Promoter and enhancer across several cell lines RegulomeDB: Minimal TF binding evidence	100.00	0.00	0.00
	rs76394293 (chr11:17372388)	Insulin, Glucose	A	Fine-mapping Type 2 Diabetes	Missense (*B7H6*)	rs148825479 rs11024270 rs12269839 rs147111794	HaploReg: Associated rs11024270, rs12269839, rs147111794 promoter and enhancers across multiple cell lines RegulomeDB: Minimal TF binding evidence for index and associated variants rs11024270, rs12269839, rs147111794	0.00	44.61	55.39
	rs1939120 (chr11:64537243)	Smoking, Hematocrit	A	Replication HDL	Intergenic (*ARL2BPP7/ SLC22A11*)		HaloReg: Promoter and enhancer across multiple cell lines RegulomeDB: Likely to affect TF binding and linked to expression of a gene target SCAN: eQTL for multiple genes in YRI cell line	19.37	80.59	0.05
	rs17376366 (chr12:20339790)	Stroke, Creatinine	G	Replication Diastolic Blood Pressure	Intergenic (*LOC100506393/ PDE3A*)	rs71463092	HaploReg: Index variant *PDE3A* eQTL in GTEx skeletal muscle	99.51	0.48	0.02
	rs115487129 (chr12:89395852)	Smoking, Alcohol	C	Fine Mapping Systolic Blood Pressure	Intergenic (DUSP6/ POC1B)		HaploReg: Enhancer and promoter in multiple cell lines RegulomeDB: Minimal TF binding evidence	2.90	20.14	76.96
*	rs7139221 (chr12:110853890)	Insulin, Smoking	A	Fine-mapping Myocardial Infarction	Intronic (*CCDC63*)	rs61944267 rs112189206 rs113945414	HaploReg: Index variant is *ARPC3*, *TCTN1*, *VPS29* eQTL for BRCA tissue RegulomeDB: Index and associated variant rs113945414 likely to affect TF binding; minimal TF binding evidence for associated rs61944267 and rs112189206 SCAN: Index variant eQTL in two genes in YRI cell line	0.00	9.98	90.02
	rs10774711 (chr12:113697994)	SBP, MI	A	Replication QT Interval	Intergenic (*LOC100506452/ LOC100506465*)	rs28723444 rs28519077 rs28578425 rs12297352 rs4767114 rs4767115 rs4767116 rs7978617 rs2384312 rs2384313	HaploReg: Index and associated variants have strong to weak enhancer evidence RegulomeDB: Index and associated variants rs28723444, rs28519077, rs28578425, rs4767114, rs4767115, rs4767116, rs7978617, rs2384312, rs2384313) have minimal evidence of TF binding	17.01	47.60	35.39
	rs60136502 (chr15:90965307)	Insulin, Total Cholesterol	C	Fine-mapping Type 2 Diabetes	Intergenic (*RCCD1/PRC1*)		HaploReg: Weak enhancer RegulomeDB: minimal TF binding evidence	2.39	61.58	36.03
	rs11865790 (chr16:47399623)	Alcohol, CRP	G	Replication Fasting Glucose	Intronic (*ITFG1*)	rs13335171 rs7197586 rs113161991 rs7202716 rs73543108 rs73543111 rs16945353 rs11860257	HaploReg: Index and associated variants weak enhancers; Associated rs7202716 and rs11860257 eQTLs of Hs.42217 in YRI cell lines RegulomeDB: Index and associated variants rs13335171, rs113161991, rs73543108, rs73543111 minimal TF binding evidence SCAN: Index and associated variants rs7202716, rs16945353, rs11860257 eQTLs for one to two genes in YRI cell line	96.58	3.42	0.00
	rs8058543 (chr16:53096347)	Heart Arterial Surgery, MI	A	Replication Two Hour Glucose Challenge	Intronic (*CHD9*)		HaploReg: Weak to strong enhancer RegulomeDB: Minimal TF binding evidence SCAN: eQTL for genes in CEU cell line	34.84	43.71	21.46
*	rs2926143 (chr16:64224173)	Hypertension, Smoking	G	Replication Diastolic Blood Pressure	Intergenic (*RPS15AP34/ LOC729217*)	rs1016891 rs889424	HaploReg: Associated rs889424 weak enhancer Regulome DB: Index and associated variants have minimal TF binding evidence	62.47	37.31	0.22
	rs4260044 (chr16:85238638)	BMI, CRP	A	Replication HDL, Replication Triglycerides	Intergenic (*LOC100506467/ LINC00311*)		HaploReg: Evidence of weak and strong enhancer across different cell lines. RegulomeDB: Minimal TF binding evidence SCAN: eQTL for two genes in YRI cell line	30.88	69.09	0.03
*	rs4334353 (chr17:55374378)	Smoking, MI	A	Replication HDL	Intergenic (*HLF*)	rs9911896 rs11079163 rs11079164 rs28619477	HaploReg: Index and associated rs9911896, rs11079163, rs11079164 weak enhancers RegulomeDB: Minimal TF binding evidence for associated variants SCAN: Index and associated rs9911896, rs11079163, rs11079164 eQTLs for several genes in YRI cell line	90.23	9.69	0.08

Abbreviations: body mass index (BMI), C-reactive protein (CRP), coded allele (CA), direction of effect (DE), high density lipoprotein cholesterol (HDL-C), expression quantitative trait locus (eQTL), linkage disequilibrium (LD), low density lipoprotein cholesterol (LDL-C), myocardial infarction (MI), systolic blood pressure (SBP), transcription factor (TF)

We also estimated local genetic ancestry at these 38 PheWAS-identified loci given that African Americans are admixed, with varying proportions of African, European, and other ancestral alleles throughout the genome (Table 5). Consistent with reported global estimates of African and European ancestry proportions [56–59], PAGE African Americans have on average 78.8% African ancestry and 21.1% European ancestry for Metabochip variants (S1 Fig). For ancestry proportions at specific PheWAS-identified loci, we found that the majority of the annotated SNPs, such as SNPs within *CELSR2* for example, were truly admixed and are consistent with global proportions (S2 Fig). However, there were loci where ancestral proportions substantially deviated from global proportions. For example, PheWAS-identified loci in *DARC*, *JAZF1*, *MTMR11*, and *TLL2* have greater proportions of European ancestry than expected (S3 Fig). Conversely, regions such as PheWAS-identified *RBKS* have significantly greater proportions of African ancestry compared to global proportions (S4 Fig).

## Discussion

We conducted here a large-scale PheWAS for >5,000 African Americans using dense array data and carefully collected and curated epidemiologic data. With these data, we replicate previous GWAS findings from mostly European-descent populations as well as identify novel pleiotropic associations. Because the PAGE study and other efforts have focused or are focusing on multi-population discovery efforts [20, 50, 60–65] as well as replication, generalization, and fine-mapping of GWAS-identified signals [43, 66–77], we focus the remainder of our **Discussion** on the potential novel pleiotropic associations identified in this African American PheWAS. Potential pleiotropic common variants were identified via single SNP tests of association by the PAGE I study followed by statistical significance filtering and comparison across phenotype classes. For tests of association with consistent statistical evidence across PAGE I studies, we further characterized the PheWAS-identified variants using functional and local genetic ancestry annotations to better understand possible mechanisms or explanations underlying the evidence for pleiotropy in this population. Of the 133 PheWAS-identified findings, we bring to attention those with the most statistical and *in silico* functional evidence.

Three PheWAS-identified variants were consistently associated with two phenotype classes in two or more PAGE study sites at p<0.01, and they or their proxies were identified as possible eQTLs and were previously associated with one of the phenotype classes in GWAS: *DOCK7* rs10889334, *APOB* rs568938, and *PHACTR1* rs9349379. All three were associated with the smoking phenotype class, and none of the three have been implicated in GWAS for any of the smoking categories curated by the NHGRI-EBI GWAS Catalog nor have they been implicated in recent gene-environment studies for lipid traits [78]. The other phenotype classes represented in these associations (LDL-C, hypertension, and diabetes) all have complex relationships with smoking, and these PheWAS data do not provide a clear causal pathway that defines the potentially pleiotropic variants’ relationships with the phenotype classes or between the phenotype classes themselves.

Among those variants without evidence of previous GWAS relationships, one example of a novel and potentially pleiotropic variant is the intronic *SPARC* rs4958487-A associated with increased glucose levels and hypertension. The secreted protein acidic and rich in cysteine (*SPARC)* gene product modulates the interaction between the extracellular matrix and surrounding cells and is highly expressed in fibrotic tissues [79]. Fibrosis is a clinical feature of hypertension, and both human and animal models support a relationship between *SPARC* and type 2 diabetes pathogenesis [80, 81]. While intronic, annotation of *SPARC* rs4958487 suggests that it is a significant eQTL in tibial artery (GTEx p = 7.0x10^-11^) and coronary artery (GTEx p = 5.0x10^-7^) tissues among others, with the A allele associated with higher *SPARC* expression compared with the G ancestral allele. Local genetic ancestry estimates for this locus suggest no deviations from expected proportions of European and African ancestry at this locus. Although *SPARC* rs4958487 has not yet been associated with any phenotype (including glucose or hypertension) at p<10^−8^ in the NHGRI-EBI GWAS Catalog, it was included on the Metabochip genotyping array for replication based on early meta-analyses of mean platelet volume in European-descent populations at p<1.0x10^-3^ [82–84]. To our knowledge, the present PheWAS in African Americans provides the first statistical and *in silico* evidence for pleiotropy for this locus, which has already been noted as likely pleiotropic based on its possible roles in type 2 diabetes, obesity, cardiovascular disease, bone strength, tendinopathies, and cancers [81, 85].

Among the annotations examined for these PheWAS-identified associations, local genetic ancestry was among the least informative. Genetic ancestry and admixture are widely recognized as useful markers of human migration [56, 58] and disease associations [86], including potential genetic interactions [87]. Here we note several PheWAS-identified variants with fewer (*DARC* “Duffy” locus, *JAZF1* rs216922, *MTMR11* rs2205303, and *TLL2* rs94208) or more (*RBKS* locus) African-derived alleles than expected. While some have interpreted deviations such as those likely to be due to natural selection since admixture [88], recent large-scale studies have suggested that most local ancestry deviations are due to chance [89].

The present study has several limitations as well as strengths. A major limitation of this and other PheWAS is sample size and power for any individual test of association, a limitation compounded by the multiple testing penalty. An ideal PheWAS would be one conducted in a large sample size of hundreds of thousands of uniformly genotyped (or sequenced) and phenotyped participants. The PAGE I study PheWAS represents a collaboration across several, independent epidemiologic cohorts each genotyped on the Metabochip, necessitating a strategy that emphasized within-study tests of association and across-study patterns of consistent results. The phenotype class assignments made here, while facilitating the within and across-study comparisons, were based mostly on study data labels interpreted by human curators rather than formal statistical examination of the phenotypic data. As a result, some correlated phenotypes were considered separate phenotype classes rather than a single large class. It is unclear, however, how to best classify the multiply-related phenotypes given that the phentoypic correlations are imperfect and the current GWAS-based evidence of overlapping but not completely identical genetic architectures for many of the phenotypes considered here.

A second major limitation of this and other PheWAS is interpretation of the observed associations. These data only include genetic variants targeted by the Metabochip [11, 12], a fixed-content array of GWAS-identified variants and fine-mapping regions from cardio-metabolic studies of mostly European populations. It is likely that other population-specific and trans-population variants not assayed here are associated with many of the phenotypes tested. For the significant associations identified in the present study, a PheWAS-identified association can be interpreted as evidence of true pleiotropy, true comorbidity, or confounding, among others [90]. The PAGE I study PheWAS-identified associations involving the phenotype class smoking illustrate this major limitation: LDL-C is associated with smoking, and the genetic variants are associated with both phenotype classes. It may be that these PheWAS results are highlighting the correlation between phenotype classes, revealing a novel causal pathway, or representing confounding. These PheWAS results could also be due to chance. Further statistical (e.g., independent statistical replication, mediation analysis, effect modification) and functional data will be required to properly interpret complex PheWAS associations.

While we acknowledge that this PheWAS has major limitations, it also has considerable strengths that complement other reported PheWAS. This PheWAS was conducted in African Americans using all Metabochip variants and phenotypes available whereas some previous PheWAS were conducted in European Americans or using specific variants or class of variants and/or a limited set of phenotypes [90–96]. The few genome-wide, phenome-wide reported PheWAS are based on clinical data extracted from electronic health records (EHRs) [8, 97, 98]. EHR-based PheWAS rely on structured phenotype data such as International Classification of Diseases codes (ICDs, otherwise known as billing codes) and laboratory values. While EHR data represent real-world clinical phenotypes, these data are not uniformly collected across all patients and are associated with known and unknown biases [99]. Also, EHR PheWAS have yet to consider unstructured exposure, behavioral, or lifestyle variables, which are known to be highly relevant to human health and disease risk but are notoriously difficult to extract from clinical free text [99]. The PAGE study is the first to introduce exposure, behavioral, and lifestyle data to the PheWAS landscape, and results suggest these variables may be relevant in describing the complex genetic architecture of traits and disease risk in humans. These results also provide useful data towards Mendelian randomization studies, which aim to use instrument variables to establish causal relationships. The ideal instrument variable is free of pleiotropy; thus, PheWAS could serve as a test of this important assumption of Mendelian randomization [100].

## Conclusions

Our work reinforces the potential of PheWAS in epidemiologically collected, diverse populations. We confirm known genetic associations as well as identify potentially pleiotropic common variants across the genome in African Americans. These data reveal complex genetic relationships between common, complex disorders and, in some cases, exposures as-of-yet undetected in univariate analyses common in GWAS, underscoring the need for phenotype-wide studies to better understand the multiple dimensions of genotype-phenotype relationships in humans.

## Methods and materials

### PAGE study sites: Designs and populations

Summary descriptions for each PAGE study site are presented in Table 1. All study protocols were approved by Institutional Review Boards at their respective study sites (S2 File).

#### Causal Variants Across the Life Course (CALiCo) and the Atherosclerosis Risk in Communities (ARIC) study

CALiCo is a consortium of six demographically diverse population-based studies comprising of 58,000 men and women ranging in age from childhood to older adulthood and a central laboratory. The ARIC study is one of the six studies included in CALiCo and is a multi-center prospective investigation of atherosclerotic disease in a predominantly bi-racial population. European American and African American men and women aged 45–64 years at baseline were recruited from four communities: Forsyth County, North Carolina; Jackson, Mississippi; suburban areas of Minneapolis, Minnesota; and Washington County, Maryland m. A total of 15,792 individuals participated in the baseline examination in 1987–1989, with follow-up examinations in approximate 3-year intervals, during 1990–1992, 1993–1995, and 1996–1998. After the institutional review board at every participating university approved the ARIC Study protocol, written informed consent was obtained from each participant. A subset of ARIC participants was selected for genotyping and inclusion in these PAGE analyses. Data dictionaries for ARIC are available on their website (https://sites.cscc.unc.edu/aric/) as well as the database of Genotypes and Phenotypes (dbGaP) [101].

#### Multiethnic cohort (MEC)

The MEC is a population-based prospective cohort study consisting of 215,251 men and women, and comprises mainly five self-reported racial/ethnic populations: African Americans, Japanese Americans, Latinos, Native Hawaiians and European Americans [14]. The MEC was designed to provide prospective data on exposures and biomarkers potentially involved in cancer initiation and progression across groups with distinct cultural and dietary patterns. Between 1993 and 1996, adults between 45 and 75 years old were enrolled by completing a 26-page, self-administered questionnaire asking detailed information about dietary habits, demographic factors, level of education, personal behaviors, and history of prior medical conditions (e.g. diabetes). Between 1995 and 2004, blood specimens were collected from ~67,000 MEC participants at which time a short questionnaire was administered to update certain exposures and collect current information about medication use. Study protocols and consent forms were approved by the institutional review boards at all participating institutions. A subset of MEC participants were selected for genotyping and inclusion in these PAGE analyses. Data dictionaries for MEC (https://www.uhcancercenter.org/mec) are available in dbGaP.

#### Women’s health initiative (WHI)

WHI is a long-term national health study that focuses on strategies for preventing heart disease, breast and colorectal cancer and fracture in postmenopausal women. A total of 161,838 women aged 50–79 years old were recruited from 40 clinical centers in the US between 1993 and 1998 [102]. WHI consists of an observational study, two clinical trials of postmenopausal hormone therapy (estrogen alone or estrogen plus progestin), a calcium and vitamin D supplement trial, and a dietary modification trial. Trial exclusion criteria have been described previously [15]. Study protocols and consent forms were approved by the institutional review boards at all participating institutions. A subset of WHI women were selected for genotyping and inclusion in these PAGE analyses. Data dictionaries for WHI are available on their website (https://www.whi.org/researchers/data/WHIStudies/StudySites/Pages/home.aspx) as well as dbGaP.

### Metabochip content and genotyping

The Metabochip has SNPs selected as GWAS replication targets for cardio-metabolic traits as well as SNPs in fine mapping regions around target SNPs [12]. The remaining SNPs on Metabochip include coverage of the HLA region, SNPs associated at genome wide significance with any human trait from the NHGRI GWAS catalog at the time of chip development, mitochondrial SNPs, SNPs on the X and Y chromosomes (not used in this study), and a series of “wild card” SNPs. Further details of this chip are available at the following URL: http://www.sph.umich.edu/csg/kang/Metabochip/.

Full Metabochip genotyping and quality control details are available in Buyske et al. [11]. Briefly, DNA samples were genotyped at the Human Genetics Center of the University of Texas-Houston (ARIC), the University of Southern California Genomics Core (MEC), and the Translational Genomics Research Institute (TGen) (WHI). Ninety HapMap YRI (Yoruba in Ibadan, Nigeria) samples were genotyped in each of the three sites for cross-site quality control. Genotypes were called separately for each PAGE study site at the PAGE Coordinating Center under a common protocol, using both the Genome Studio GenCall 2.0 algorithm as well the GenoSNP genotyping algorithm [103], which is a sample-based approach for capturing some of the rarer genotypes represented on Metabochip. Discordance between the results of the two algorithms were used as a quality control filter. A total of 0.9% of samples were removed based on sample quality control measures. A total of 14,328 (7.3%) SNPs was considered technical failures because of the GenCall or cluster separation score, call rate, Mendelian error rate, replication error rate, or deviation from Hardy Weinberg Equilibrium. An additional 5,248 (2.7%) SNPs were not used in this study because the probe sequence matched poorly to the reference genome. Identification of related individuals were identified using PLINK [104] and the calculations of identity by descent (IBD) for all pairs, up to 2^nd^ degree relatives. For pairs identified as related, one from each pair was dropped out of further analysis based on which individual had the higher call rate. Overall, 5,897 samples and 161,097 SNPs on the Metabochip passed the quality control criteria of the PAGE I study. A total of 144,740 of these SNPs passed the present study allele frequency threshold (1%).

To adjust for population stratification across study sites, principal components were determined separately for each PAGE I study using the smartpca package of the Eigensoft software [105]. The first two principal components were used as covariates in all analyses. Full analysis details for the ancestry adjustments are also available in Buyske et al. [11].

### Genetic tests of association

All tests of association were performed separately for each PAGE I study site in PLINK [104] and adjusted for the first two principal components and sex (except for the women only WHI). A total of 144,740 SNPs were used in the PheWAS for 273 phenotypes. S1 Table lists the 273 phenotypes used in this study. Linear or logistic regression was performed for continuous or categorical dependent variables, respectively, assuming an additive genetic model (0, 1, or 2 copies of the coded allele). For variables with multiple categories, binning was used to create new variables of the form “A versus not A” for each category, and logistic regression was used to model the new binary variable. Linear regressions were repeated following a y to log (y+1) transformation of the response variable with +1 added to all continuous measurements before transformation to prevent variables recorded as zero from being omitted from analysis. The total numbers of associations calculated for this PheWAS where the coded allele frequency was greater than 1% were ARIC 22x10^6^, MEC 8x10^6^, and WHI 26x10^6^. Data were visualized using PhenoGram [106].

### Phenotype Class Matching

All 273 individual PAGE study phenotypes were grouped into categories within sites and then grouped into categories across sites regardless of genetic association. As an example of within study collapsing, WHI had four separate phenotypic measurements related to diabetes, including “Diabetes ever (Y/N)” and “treated diabetes (Y/N)”, all binned together in the same phenotype class. Across PAGE, specific phenotypes clearly were collected for more than one study site, such as for the phenotype “Hemoglobin”. Other groups of phenotypes that fell within similar phenotypic domains but were not represented in the same form across PAGE study sites (e.g., hormone use, smoking) were also collapsed into phenotype classes. Phenotype classes were developed by one curator, and a second curator reviewed the resultant phenotypes and phenotype classes for consistency and accuracy. Neither curator used genetic association results in the development or review of the phenotype classes. The end result was a total of 30 phenotype classes.

### Permutation testing

To determine an empirically derived p-value threshold, we used permutation testing. PheWAS is exploratory and thus incurs a substantial multiple hypothesis testing burden depending on the number of associations being calculated. Dependent on individual PAGE study, a Bonferroni correction would have resulted in an adjusted p-value threshold between ~4x10^-9^ and ~7x10^-9^ (S1 File). Bonferroni correction is not suitable for this and other PheWAS as there are correlations between phenotypes as well as correlations between SNPs (i.e., linkage disequilibrium). Therefore, the multiple associations of this PheWAS cannot be considered independent. To determine an empirical p-value threshold, we took a two-step approach. The first step was to permute the data within each study separately (ARIC, MEC, WHI):

1. Randomize the association between the genotype matrix and the phenotype matrix 1000 times, generating 1000 individual datasets.
  - This preserved the relationships between genotypes
  - This preserved the relationships between phenotypes
2. Perform PheWAS—comprehensive tests of association between all the phenotypes and genotypes—for each of the 1000 permuted datasets
  - Output: Results for 1000 permuted PheWAS datasets

The second step was to determine how often SNP-phenotype associations were significant across two or more studies by chance alone (in the permuted null data). These results were then compared to the results from the unpermuted data. Our definition of replication was two or more studies with an association for the same phenotype class at a specific p-value threshold, and in S1 File we present results across 1000 permutations at various p-value thresholds. When requiring replication at any of our p-value thresholds, any single permuted data set did not have a total number of results equal to or greater than the total number of results in the unpermuted data, indicating that requiring replication and using a p-value threshold of 0.01 would allow us to explore the data while still maintaining a stringent-enough threshold to reduce our type-1 error rate. As we wanted to explore pleiotropy, we wanted to explore how many different phenotype classes we would expect for a single SNP by chance alone. Thus, we also compared the results of the permuted data versus the non-permuted data, when requiring replication for any single SNP result *and* more than one phenotype-class at different p-value thresholds. S1 File also presents results across 1000 permutations, requiring replication for each individual phenotype class, for more than one phenotype class at various p-value thresholds. At a p-value threshold of 0.01, we found only three permuted data sets with more SNPs associated with more than one phenotype class, compared to the 188 results of the non-permuted data. It is important to note that within the non-permuted data the 188 results were further refined, we removed any results that did not have the same direction of effect across studies.

### Functional annotation

For each independent PheWAS-identified variant as well as SNPs in linkage disequilibrium (r^2^≥0.80), we annotated individual variants using HaploReg v4.1 [53], RegulomeDB v1.1 [54], and the SNP and CNV Annotation Database (SCAN) [55] (http://www.scandb.org/newinterface/index.html). HaploReg v4.1 [53] (http://www.broadinstitute.org/mammals/haploreg/haploreg.php) annotates SNPs with ENCODE and GENCODE, GTEx [107], and NHGRI-EBI GWAS Catalog data [108]. We supplemented GWAS annotations using the more recent (2019-June 20) version of the NHGRI-EBI GWAS Catalog. RegulomeDB [54] annotates variants based on evidence for transcription factor binding. SCAN is a database that provides summary information from eQTL experiments, mapping HapMap SNPs to gene expression in European Americans from UT, USA (CEU) and Yoruba people from Ibadan, Nigeria (YRI). The database provides a list of genes showing local and distant associations to the SNP in these two HapMap populations along with p-values calculated using quantitative trait linkage disequilibrium test (QTDT) method. The database also provides functional summary information available from other databases as well as other GWAS summary information for the SNPs used for annotation.

### Genetic ancestry

We estimated both global and local genetic ancestry for all PAGE African Americans in this study. Global ancestry was estimated using ~196k SNPs on the Metabochip array and the ADMIXTURE software assuming K = 2 populations [109]. Although this test was unsupervised, HapMap YRI samples were included and a 5-fold cross validation was used to ensure accuracy. Local estimates of ancestry were calculated using LAMP-LD [110] for ~175,600 SNPs after LD pruning in PLINK [104]. Phased haplotypes for CEU and YRI reference samples from the 1000 Genomes Project were used. We calculated local ancestry using a sliding window of 50 SNPs (200 kb) and 10 states per SNP per recommended by the LAMP-LD manual for maximal accuracy and minimal computational time [110].

## Supporting information

S1 Table List of phenotypes included in the Population Architecture using Genomics and Epidemiology (PAGE) I phenome-wide association study (PheWAS), by PAGE study. Given are the abbreviations and brief descriptions of phenotypes available for the PAGE I study PheWAS in African Americans by PAGE study.
(XLSX)

S2 Table All PheWAS tests of association for the Population Architecture using Genomics and Epidemiology (PAGE) I study in African Americans. We tested 144,740 SNPs assayed on the Metabochip with up to 273 phenotypes available for 5,897 African American participants from three participating PAGE I studies: Atherosclerosis Risk in Communities (ARIC); Multiethnic Cohort (MEC); and the Women’s Health Initiative (WHI). A total of 5,424 tests of association were significant at p<0.01 in two or more PAGE studies and in the same direction for the same phenotype. For each of these significant tests of association, we give the SNP (rs number, chromosome, position), the number of PAGE studies with significant results (at p<0.01), the names of the PAGE studies tested for each phenotype listed regardless of significance, the phenotypes tested (short names, long names, and phenotype classes), the statistics (p-values, betas, standard errors), sample size, coded allele and allele frequency, and reason why the variant was assayed by the Metabochip.
(XLSX)

S3 Table Significant PheWAS tests of association for the Population Architecture using Genomics and Epidemiology (PAGE) I study in African Americans. After significance threshold filtering and phenotype class binning, we identified 133 SNPs associated with two or more distinct phenotype classes with the same direction of effect within a given phenotype class. For each of these PheWAS-identified variants, we give the SNP (rs number, chromosome, position), the number and name of PAGE studies with significant results, the phenotypes associated, the statistics (p-values, betas, standard errors), sample size, coded allele and allele frequency, reason why the variant was assayed by the Metabochip, and nearest genes.
(XLSX)

S1 File Supporting text and tables for deriving the p-value threshold. (DOCX)

S2 File Individual institutional review boards that approved the current study. (DOCX)

S1 Fig Global genetic ancestry estimated for African Americans in the Population Architecture using Genomics and Epidemiology (PAGE) I study. Global genetic ancestry was estimated using ADMIXTURE (unsupervised, assuming K = 2) and all single nucleotide polymorphisms (SNPs) assayed on the Metabochip that passed quality control measures (~196K). Data from the HapMap YRI reference samples were included, representing West African ancestry, and a 5-fold cross validation was used to ensure accuracy. Plotted are the individual samples’ (x-axis) estimated global ancestry (y-axis) for the HapMap YRI reference dataset (left) and the PAGE African American Metabochip dataset (right). European ancestry is color-coded blue while African ancestry is color-coded red. Average African ancestry (European) ancestry for PAGE African Americans in this phenome-wide association study is 78.8% (21.1%).
(PNG)

S2 Fig PheWAS-identified admixed loci based on local genetic ancestry estimates. Local estimates of ancestry were calculated using LAMP-LD for ~175,600 SNPs after LD pruning in PLINK. Phased haplotypes for European (CEU) and West African (YRI) reference samples from the 1000 Genomes Project were used. Local ancestry was calculated using a sliding window of 50 SNPs (200 kb) and 10 states per SNP per recommended by the LAMP-LD documentation for maximal accuracy and minimal computational time. For each PheWAS-identified single nucleotide polymorphism (SNP), we characterized the estimated genetic ancestry as number of (y-axis) copies of African- or European-derived allele (x-axis). A locus was considered “admixed” if the proportion of African-derived alleles was consistent with global genetic ancestry estimates (78.8% West African, S1 Fig). Examples of PheWAS-identified admixed loci include ***a) B7H6-NCR3LG1* rs76394293 *b) PHACTR1* rs9349379 *c) SPARC* rs4958487 *d) CELSR2* rs7528419, rs12740374, rs660240, rs62931, rs646776**.
(TIFF)

S3 Fig PheWAS-identified European-derived loci based on local genetic ancestry estimates. Local estimates of ancestry were calculated using LAMP-LD for ~175,600 SNPs after LD pruning in PLINK. Phased haplotypes for European (CEU) and West African (YRI) reference samples from the 1000 Genomes Project were used. Local ancestry was calculated using a sliding window of 50 SNPs (200 kb) and 10 states per SNP per recommended by the LAMP-LD documentation for maximal accuracy and minimal computational time. For each PheWAS-identified single nucleotide polymorphism (SNP), we characterized the estimated genetic ancestry as number of (y-axis) copies of African- or European-derived allele (x-axis). A locus was considered “admixed” if the proportion of African-derived alleles was consistent with global genetic ancestry estimates (78.8% West African, S1 Fig); else, it was classified as either “African-derived” or “European-derived.” Examples of PheWAS-identified European-derived loci include ***a) DARC* “Duffy” locus *b) JAZF1* rs216922 *c) TLL2* rs94208 *d) MTMR11* rs2205303**.
(TIF)

S4 Fig PheWAS-identified African-derived locus *RBKS* based on local genetic ancestry estimates. Local estimates of ancestry were calculated using LAMP-LD for ~175,600 SNPs after LD pruning in PLINK. Phased haplotypes for European (CEU) and West African (YRI) reference samples from the 1000 Genomes Project were used. Local ancestry was calculated using a sliding window of 50 SNPs (200 kb) and 10 states per SNP per recommended by the LAMP-LD documentation for maximal accuracy and minimal computational time. For each PheWAS-identified single nucleotide polymorphism (SNP), we characterized the estimated genetic ancestry as number of (y-axis) copies of African- or European-derived allele (x-axis). A locus was considered “admixed” if the proportion of African-derived alleles was consistent with global genetic ancestry estimates (78.8% West African, S1 Fig); else, it was classified as either “African-derived” or “European-derived”.
(BMP)
