# Supplementary material for: A phenome-wide association study (PheWAS) in the Population Architecture using Genomics and Epidemiology (PAGE) study reveals potential pleiotropy in African Americans
Source: PLoS One. 2019 Dec 31;14(12):e0226771. doi: 10.1371/journal.pone.0226771 (PMC6938343; doi:10.1371/journal.pone.0226771)
Supplement: S1 File — (DOCX) [file pone.0226771.s004.docx]

**S1 File. Supporting Tables for Deriving P-Value Threshold**

**Resultant Bonferroni correction if considering the number of tests of association for each individual Population Architecture using Genomics and Epidemiology (PAGE) study**

| **Study** | **# Tests of Association** | **Bonferroni corrected p-value at 0.05** |
| --- | --- | --- |
|  |  |  |
| ARIC | 22,604,861 | 2.21x10^-9^ |
|  |  |  |
| MEC | 8,378,703 | 5.97x10^-9^ |
|  |  |  |
| WHI | 25,595,069 | 1.95x10^-9^ |
|  |  |  |

**Results across 1000 permutations, requiring replication (2 or more PAGE studies with an association for the same phenotype class at a p-value threshold) compared to replication in the unpermuted data counts, for a range of p-value thresholds.**

| P-value threshold | Real Data Counts^1^ | Permuted Min^2^ | Permuted Mean^3^ | Permuted Max^4^ | # Times > unpermuted across 1000 permutations^5^ |
| --- | --- | --- | --- | --- | --- |
| 5x10^-4^ | 437 | 15 | 39 | 117 | 0 |
| 1x10^-3^ | 527 | 49 | 94 | 204 | 0 |
| 5x10^-3^ | 1877 | 1019 | 1240 | 1501 | 0 |
| 1x10^-2^ | 5424 | 3752 | 4247 | 4760 | 0 |

- - 1. The count of results in the unpermuted data, where after requiring replication, the p-value for the replicating results was less than the p-value threshold.
    2. In the permuted data: the number of replicating results for the one permuted data set with the least number of replicating results, out of all permuted datasets, with p-values less than the p-value threshold.
    3. The mean number of replicating results, across the 1000 permuted data sets, where a permuted dataset had p-values less than the p-value threshold.
    4. In the permuted data: the number of replicating results for the one permuted data set with the highest number of replications, out of all permuted datasets, with p-values less than the p-value threshold.
    5. The number of times any of the permuted data set had a number of results equal to or greater than the total number of results in the unpermuted data set with a p-value less than the p-value threshold.

**Results across 1000 permutations, requiring replication, *and* two or more distinct phenotype classes for the same SNP, compared to the same requirements for the unpermuted data, at different p-value thresholds.**

| P-­value threshold | Real Concomitant Data Counts^1^ | Permuted Min^2^ | Permuted Mean^3^ | Permuted Max^4^ | # Times > unpermuted across 1000 permutations^5^ |
| --- | --- | --- | --- | --- | --- |
| 5x10^-4^ | 9 | 0 | <1 | 13 | 1 |
| 1x10^‐3^ | 13 | 0 | <1 | 13 | 0 |
| 5x10^‐3^ | 62 | 2 | 12 | 143 | 2 |
| 1x10^‐2^ | 188 | 53 | 96 | 252 | 3 |

1. The count of concomitant results, in the unpermuted data, with a p-value less than the p-value threshold.
2. In the permuted data: the number of results for the permuted data set with the least number of replicating results, out of all of the permuted data sets, for concomitant results.
3. The mean number of results, across the 1000 permuted data sets, with a p-value less than the p-value threshold, across more than one study, for concomitant results.
4. In the permuted data: the number of results for the permuted data set with the highest number of replicating results, out of all the permuted data sets, for concomitant results.
5. The number of times any of the permuted data had a number of results equal to or greater than the total number of results in the unpermuted data with a p-value less than the p-value threshold, for concomitant results.
