# Supplementary material for: A phenome-wide association study (PheWAS) in the Population Architecture using Genomics and Epidemiology (PAGE) study reveals potential pleiotropy in African Americans
Source: PLoS One. 2019 Dec 31;14(12):e0226771. doi: 10.1371/journal.pone.0226771 (PMC6938343; doi:10.1371/journal.pone.0226771)
Supplement: S2 File — (DOCX) [file pone.0226771.s005.docx]

**S2 File 2. Individual Institutional Review Boards that approved the Population Architecture using Genomics and Epidemiology (PAGE) I study.** Listed here are the individual Institutional Reviews Boards that approved the PAGE I studies included in this phenome-wide association study. The ARIC study has been approved by Institutional Review Boards (IRBs) at all participating institutions: University of North Carolina at Chapel Hill IRB, Johns Hopkins University IRB, University of Minnesota IRB, and University of Mississippi Medical Center IRB. The Multiethnic Cohort (MEC) has been approved by IRBs at the University of Hawaii Cancer Center and Keck School of Medicine, University of Southern California. Research using the Women’s Health Initiative (WHI) has been approved by the Fred Hutchinson Cancer Research Center IRB.
