# Supplementary figures and images for: A phenome-wide association study (PheWAS) in the Population Architecture using Genomics and Epidemiology (PAGE) study reveals potential pleiotropy in African Americans
Source: PLoS One. 2019 Dec 31;14(12):e0226771. doi: 10.1371/journal.pone.0226771 (PMC6938343; doi:10.1371/journal.pone.0226771)

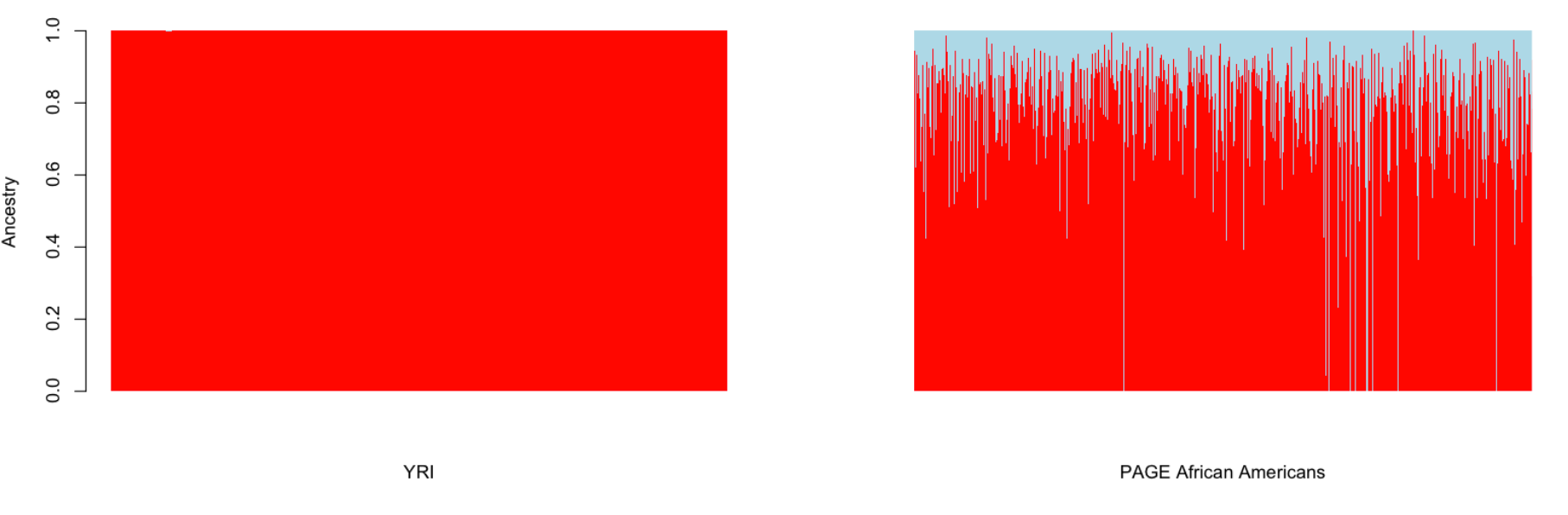

Supplement: S1 Fig — Global genetic ancestry was estimated using ADMIXTURE (unsupervised, assuming K = 2) and all single nucleotide polymorphisms (SNPs) assayed on the Metabochip that passed quality control measures (~196K). Data from the HapMap YRI reference samples were included, representing West African ancestry, and a 5-fold cross validation was used to ensure accuracy. Plotted are the individual samples’ (x-axis) estimated global ancestry (y-axis) for the HapMap YRI reference dataset (left) and the PAGE African American Metabochip dataset (right). European ancestry is color-coded blue while African ancestry is color-coded red. Average African ancestry (European) ancestry for PAGE African Americans in this phenome-wide association study is 78.8% (21.1%). (PNG) [file pone.0226771.s006.PNG]

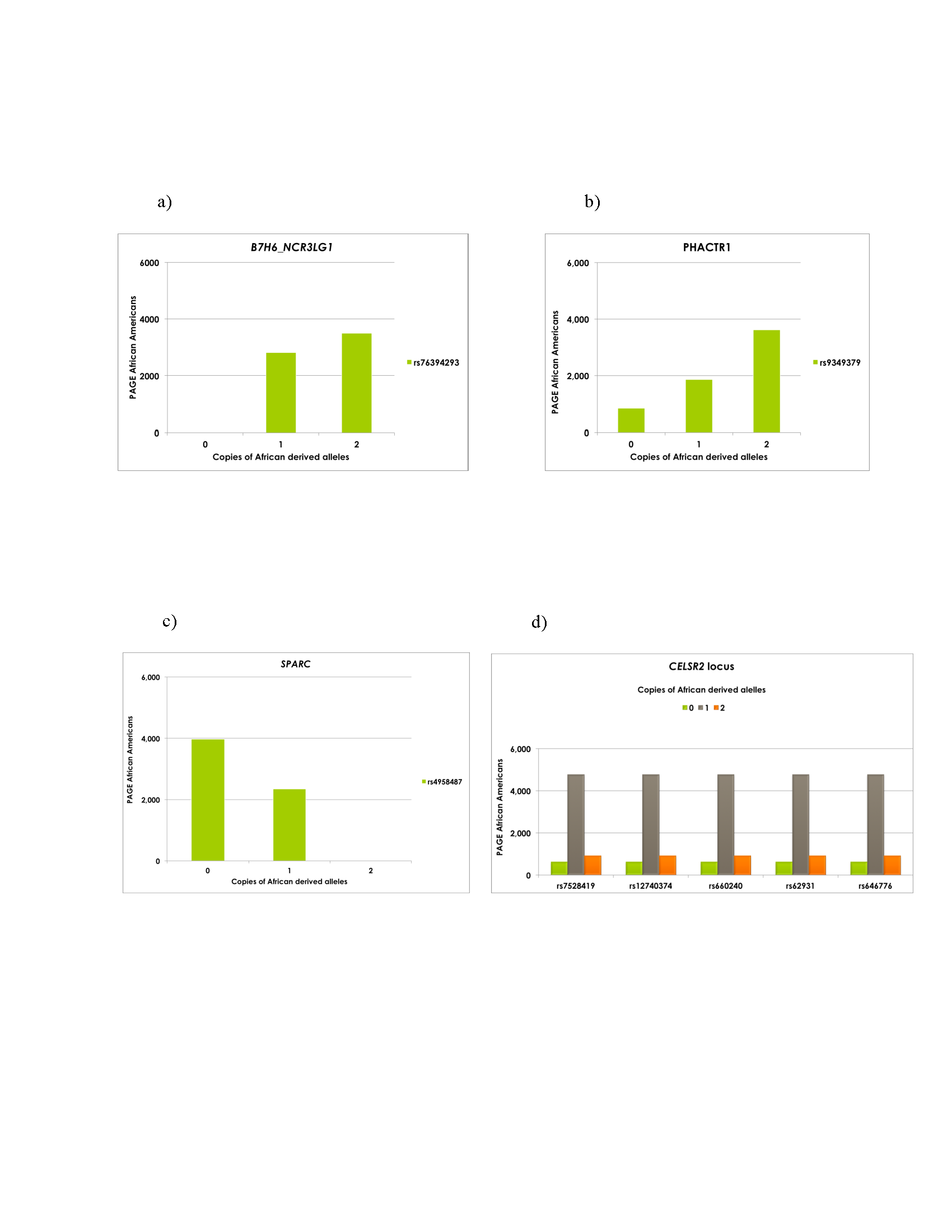

Supplement: S2 Fig — Local estimates of ancestry were calculated using LAMP-LD for ~175,600 SNPs after LD pruning in PLINK. Phased haplotypes for European (CEU) and West African (YRI) reference samples from the 1000 Genomes Project were used. Local ancestry was calculated using a sliding window of 50 SNPs (200 kb) and 10 states per SNP per recommended by the LAMP-LD documentation for maximal accuracy and minimal computational time. For each PheWAS-identified single nucleotide polymorphism (SNP), we characterized the estimated genetic ancestry as number of (y-axis) copies of African- or European-derived allele (x-axis). A locus was considered “admixed” if the proportion of African-derived alleles was consistent with global genetic ancestry estimates (78.8% West African, S1 Fig). Examples of PheWAS-identified admixed loci include a) B7H6-NCR3LG1 rs76394293 b) PHACTR1 rs9349379 c) SPARC rs4958487 d) CELSR2 rs7528419, rs12740374, rs660240, rs62931, rs646776. (TIFF) [file pone.0226771.s007.tiff]

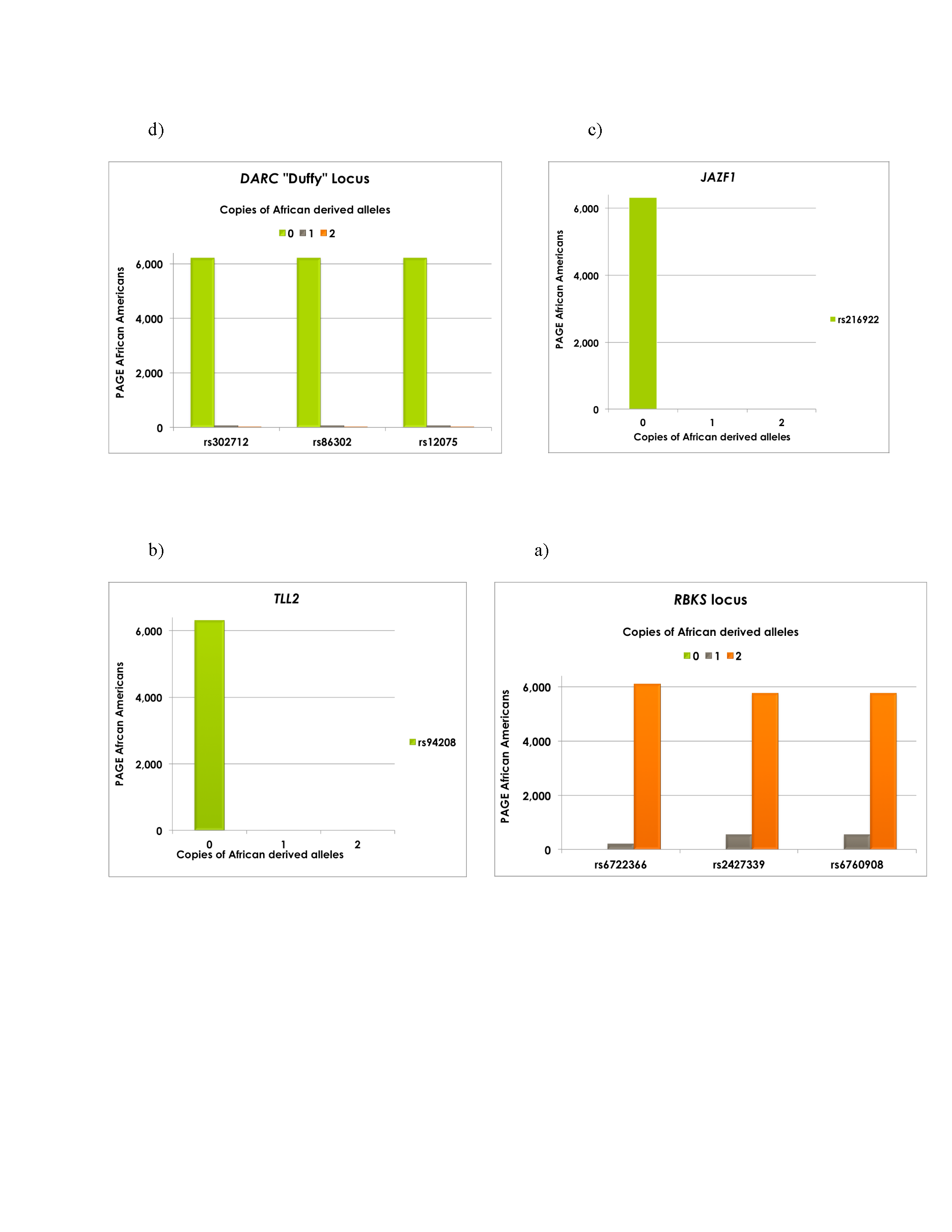

Supplement: S3 Fig — Local estimates of ancestry were calculated using LAMP-LD for ~175,600 SNPs after LD pruning in PLINK. Phased haplotypes for European (CEU) and West African (YRI) reference samples from the 1000 Genomes Project were used. Local ancestry was calculated using a sliding window of 50 SNPs (200 kb) and 10 states per SNP per recommended by the LAMP-LD documentation for maximal accuracy and minimal computational time. For each PheWAS-identified single nucleotide polymorphism (SNP), we characterized the estimated genetic ancestry as number of (y-axis) copies of African- or European-derived allele (x-axis). A locus was considered “admixed” if the proportion of African-derived alleles was consistent with global genetic ancestry estimates (78.8% West African, S1 Fig); else, it was classified as either “African-derived” or “European-derived.” Examples of PheWAS-identified European-derived loci include a) DARC “Duffy” locus b) JAZF1 rs216922 c) TLL2 rs94208 d) MTMR11 rs2205303. (TIF) [file pone.0226771.s008.tif]

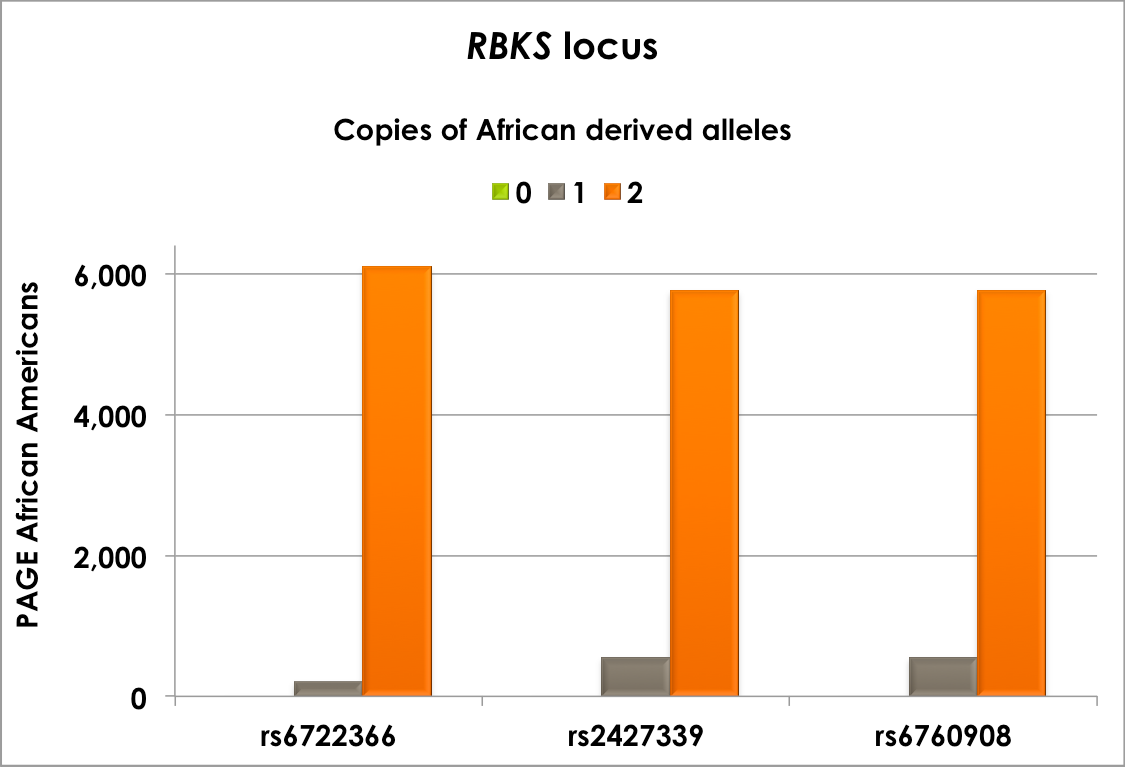

Supplement: S4 Fig — Local estimates of ancestry were calculated using LAMP-LD for ~175,600 SNPs after LD pruning in PLINK. Phased haplotypes for European (CEU) and West African (YRI) reference samples from the 1000 Genomes Project were used. Local ancestry was calculated using a sliding window of 50 SNPs (200 kb) and 10 states per SNP per recommended by the LAMP-LD documentation for maximal accuracy and minimal computational time. For each PheWAS-identified single nucleotide polymorphism (SNP), we characterized the estimated genetic ancestry as number of (y-axis) copies of African- or European-derived allele (x-axis). A locus was considered “admixed” if the proportion of African-derived alleles was consistent with global genetic ancestry estimates (78.8% West African, S1 Fig); else, it was classified as either “African-derived” or “European-derived”. (BMP) [file pone.0226771.s009.bmp]
